# Synthesis of vegetable oil-based furfurylamine-derived monomer and its polymerization behaviour

**DOI:** 10.1039/d6ra05111a

**Published:** 2026-08-25

**Authors:** Jundan Chen, Yanbei Chen, Xianhong Zhang, Dong Chen, Yuhong Ma, Wantai Yang

**Affiliations:** a State Key Laboratory of Chemical Resource Engineering, Beijing University of Chemical Technology Beijing 100029 China chendong@mail.buct.edu.cn yangwt@mail.buct.edu.cn; b Beijing Engineering Research Center for the Syntheses and Applications of Waterborne Polymers, College of Materials Science and Engineering, Beijing University of Chemical Technology Beijing 100029 China; c State Key Laboratory of Chemical Engineering and Low-Carbon Technology, Department of Chemical Engineering, Tsinghua University Beijing 100084 China

## Abstract

Vegetable oils (VOs) are regarded as important bio-based raw materials due to their wide availability, low cost, renewable nature, and flexible functionalization and modification. In this work, a series of VO-based furfurylamine-derived monomers were synthesized *via* a solvent-free aminolysis reaction with 2-furfurylamine (FA), which involved a rigid furan ring and fatty amide with a flexible aliphatic chain. These monomers retain favourable renewability, degradability, and processability of bio-based feedstocks, while obtaining enhanced reactivity and rigidity from the furan moiety. As a proof of concept, the uncatalyzed aminolysis reaction between soybean oil (SO) and FA was performed at 90 °C and reached equilibrium after 72 h, giving soybean oil-based furan amide (SO–FA) with an ester group conversion of 61%. As an organocatalyst, 1,5,7-triazabicyclo[4.4.0]dec-5-ene (TBD, 1.5 wt%) could dramatically accelerate the aminolysis process, achieving 87% conversion within 3 h at 80 °C. Subsequently, a bio-based poly(soybean oil-based furan amide-*co*-maleic anhydride) (PSOFM) was prepared by copolymerization of SO–FA and MAH, and PSOFM_1_ with 56% yield and number average molecular weight (*M*_n_) of 9200 g mol^−1^ was obtained under optimized reaction conditions. Due to the participation of double bonds within the fatty acid chain during copolymerization, PSOFM copolymer with branched structure was formed, and higher unsaturation content led to increased molecular weight and broader molecular weight distribution. This work presents a facile and efficient strategy for synthesis of VO-based monomers and corresponding functional polymers from readily available biomass.

## Abstract 2

An efficient strategy converts vegetable oils into furan amide monomers, which copolymerize with maleic anhydride to yield functional bio-based copolymers.

## Graphical abstract

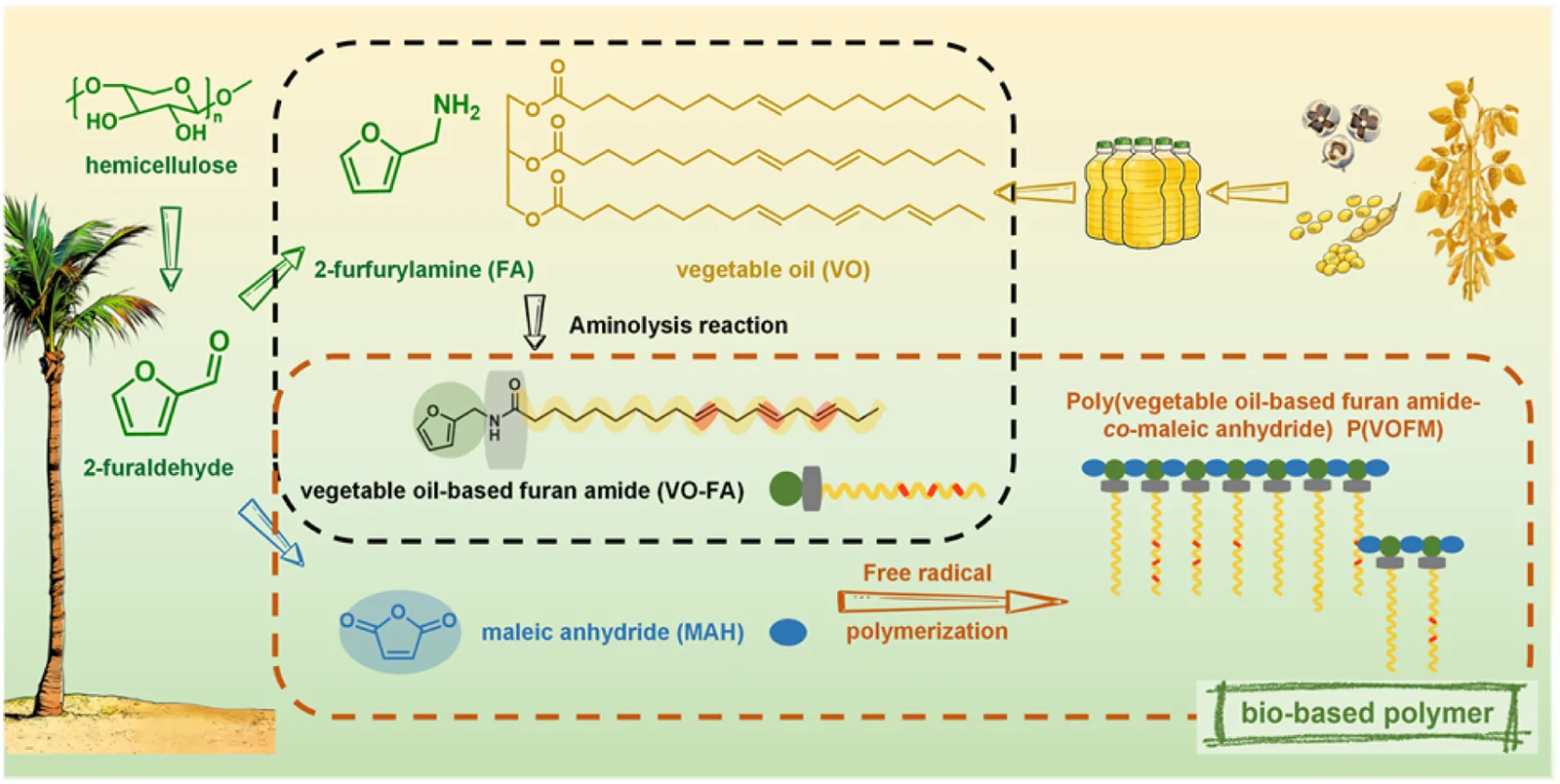

## 1. Introduction

The increasing emphasis on sustainability has driven intensive demand for eco-friendly bio-based polymer materials. These renewable materials provide viable alternatives to petroleum-based products, tackling issues like resource depletion, pollution, and greenhouse gas emissions. With their low carbon footprint and biodegradability, bio-based polymer materials efficiently support sustainable development, making them a central focus in polymer science.^1–3^

Among various bio-based resources, vegetable oils (VOs) derived from abundant crops like soybean, rapeseed, and palm are renewable and cost-effective feedstocks with low environmental impact, making them ideal for developing sustainable polymer materials.^4,5^ According to the United States Department of Agriculture (USDA), global VOs production is expected to reach 2.35 billion tons in the 2025/26 marketing year, ensuring abundant and reliable supply.^6^ Among various kinds of oil crops, global soybean production is forecast to hit a record of 428.2 million metric tons, and soybean stocks are projected to reach 125.5 million metric tons.^7^ As their production continues to expand, VOs play a significant role in both global markets and diverse applications, and industrial usage of VOs is growing steadily.^8^

As an oil mixture composed mainly of triglycerides with long-chain fatty acids, VOs possess key properties such as flexibility, hydrophobicity, and biodegradability.^9,10^ These oils can be chemically modified through epoxidation,^11,12^ ester exchange,^13,14^ and click^4^ reactions to create versatile monomers with functional groups like double bonds and epoxy groups.^15–17^ These functionalization approaches significantly broaden the application of VOs in a wide range of fields. For example, epoxidized soybean oil, acrylated VOs and their derivatives have been successfully developed and are widely used as plasticizers, adhesives, and coatings. Specifically, the vegetable oil-based polymers have potential applications in biomedical fields, environmentally friendly adhesives, phase change energy storage, elastomers, packaging materials, and functional additives.^17–20^ Demchuk *et al.*^21^ developed bio-based vinyl monomers from VOs, which can endow the as-prepared VO-based polymers with tunable hydrophobicity and refractive indices. Their homopolymers with glass transition temperatures ranging from −6 °C to 4.2 °C show great potential for eco-friendly coatings, plastics, and bio-based composites with tailored properties. Liu *et al.*^14^ designed and synthesized VO-based epoxy acrylate monomers, and stable latexes containing epoxy moieties were successfully created using these monomers. The emulsion showed excellent adhesion, water resistance and reprocessability, which make it suitable for eco-friendly adhesives and coatings.

However, conventional VO-based polymer materials often suffer from challenges of low mechanical strength and limited thermal stability, primarily due to the high flexibility of long fatty acid chains and the absence of rigid structural units. These shortcomings limit the wide application of VO-based polymer materials in cutting-edge fields. Therefore, incorporating rigid group into VO-based monomer is a key strategy to enhance performance of VO-based materials and expand their application scope.^13^ For example, Geng *et al.*^22^ reported a palm oil-based waterborne polyurethane with high solid content (51%) and low viscosity (277.3 mPa s). Notably, the incorporation of 1,6-naphthalenediol enhanced backbone rigidity, resulting in significantly improved mechanical properties (tensile strength up to 13.9 MPa, elongation up to 384%) and excellent coating performance.

As a bio-based aromatic O-heterocycle, furan ring possesses a rigid, planar structure that can effectively enhance mechanical strength and thermal stability of polymer materials.^23,24^ Hence, furan derivatives offer a promising platform to address the limitations of conventional VO-based polymers. Their incorporation into VO-based polymers *via* dynamic covalent bonds, particularly through Diels–Alder reactions with dienophiles such as maleic anhydride and maleimide, can introduce smart properties like self-healing, reprocessability and controllable degradability for the development of high-performance and sustainable materials.^13,25–27^ As a widely used building block with reactive amino group, 2-furfurylamine (FA) provides an effective way to incorporate furan rings into polymer chains through aminolysis reaction. It is reported that VO-based furan amide (VO–FA) containing furan ring and fatty acid amide structure was specially designed and synthesized *via* direct amidation of fatty acid with FA, achieving synergistic integration of flexible fatty acid chain from VO with rigid furan ring. VO–FA not only retains the renewable, degradable, and favourable processing characteristics of VO feedstock, but also incorporates the reactivity, rigidity and dynamic features imparted by furan rings.

Previous studies have explored various methods for synthesizing *N*-furfuryl amides.^20,28–30^ For instance, Yildirim *et al.*^30^ developed a reverse micelle-mediated amidation method achieving high yields (80–97%). However, this approach involved complex steps and high temperatures (up to 150 °C), and showed limited applicability toward fatty acid esters, triglycerides, and fatty acids with excessive steric hindrance. Similarly, Zheng *et al.*^28^ reported a solvent-free aminolysis method for VOs with amines under mild conditions. Specifically, the reactions with primary amines attained yields in the range of 61–85%, while those with diamines afforded yields of 82–91%. However, when applied to FA and VOs, this method suffered from low conversion rates and poor efficiency. Therefore, it is highly desired to develop a facile and efficient method for the high-yield synthesis of VO–FA under mild conditions.

In our present study, a one-pot, solvent-free, organic base catalytic aminolysis method was proposed for the facile synthesis of VO–FA, which is applicable to a wide variety of VOs and organic amines. Using soybean oil (SO) and FA as model reactants, the corresponding soybean oil-based furan amide (SO–FA) was successfully synthesized. The approach provides an environmentally friendly and highly efficient solution. Benefiting from the unique reactivity of furan ring, the as-prepared VO–FA can effectively copolymerize with maleic anhydride, resulting in the formation of a predominantly alternating copolymer poly(vegetable oil-based furan amide-*co*-maleic anhydride) (PVOFM). Using SO–FA and MAH as model reactants, resulting in copolymer poly(soybean oil-based furan amide-*co*-maleic anhydride) (PSOFM). The copolymerization behaviour of SO–FA and maleic anhydride was investigated in details. This work successfully designs and synthesizes a series of novel VO-based furfurylamine-derived monomers and the corresponding sustainable functional polymers, which greatly broaden the application scope of VO feedstock and VO-based furfurylamine-derived monomers.

## 2. Experimental

### 2.1. Chemicals and materials

Soybean oil (SO), 2-furfurylamine (FA, 99%), 1,5,7-triazabicyclo [4.4.0]dec-5-ene (TBD, ≥98%), 1,4-diazabicyclo(2.2.2)octane (DABCO, ≥98%) and tung oil (TO) were supplied by Aladdin Industrial Corporation. Palm oil (PO) and 2,2′-azobis (isobutyronitrile) (AIBN, 99%) were purchased from Macklin Reagent Co., Ltd. Maleic anhydride (MAH, >99%) was provided by Tokyo Chemical Industry (Shanghai) Development Co., Ltd. Isoamyl acetate (IPA), *n*-hexane (HEX) and tetrahydrofuran (THF) were provided by Tianjin Fuchen Chemical Reagent Factory, all these solvents are of analytical purity.

### 2.2. Synthesis of VO–FA

The products obtained from the aminolysis of vegetable oils with furfurylamine are collectively referred to as vegetable oil-based furan amide (VO–FA). Using SO as the model substrate, the as-prepared product is designated as soybean oil-based furan amide (SO–FA). To further verify the general applicability of the aminolysis method, PO and TO were also employed as feedstocks under the same reaction conditions, affording the corresponding products denoted as PO-based furan amide (PO–FA) and TO-based furan amide (TO–FA), respectively.

#### 2.2.1. Synthesis of SO–FA

**Scheme 1:**
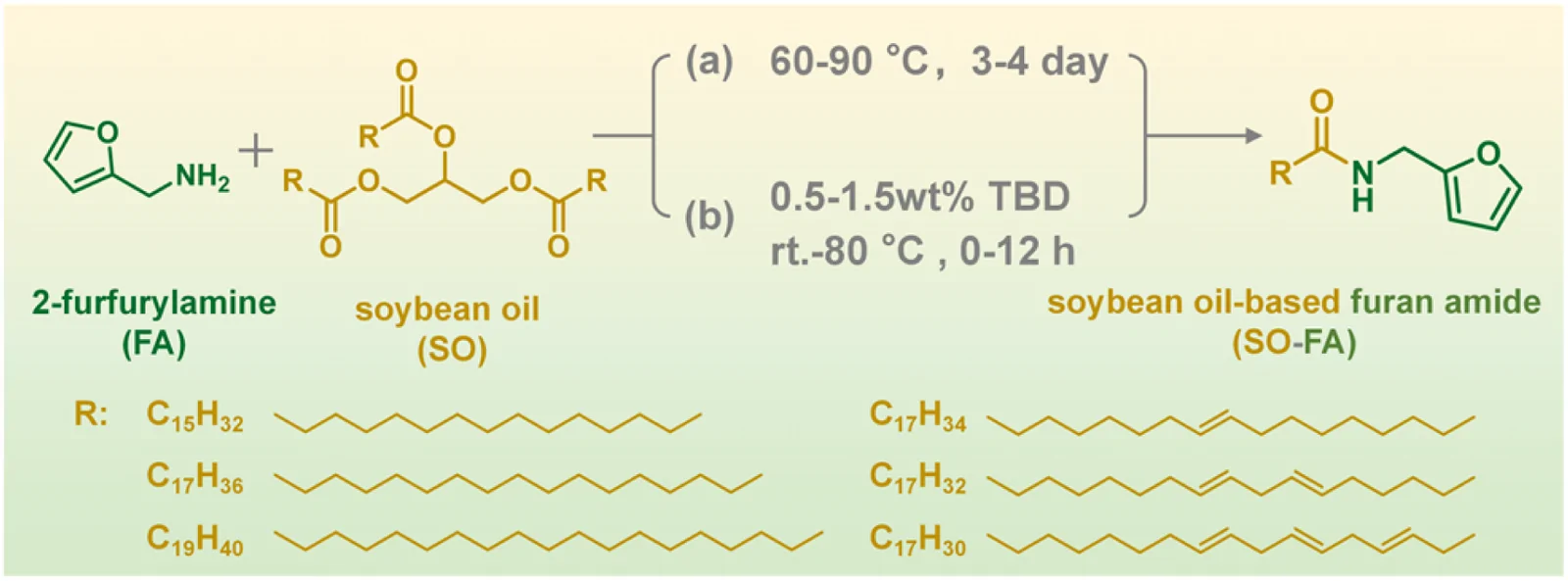
Synthetic route of SO–FA.

As depicted in Scheme 1, the synthesis of SO–FA monomer can be achieved through two distinct approaches both conducted under solvent-free conditions. Synthetic route (a) employs a catalyst-free procedure, where an increased FA loading and extended reaction time are required to achieve a high conversion of SO. In a typical procedure, FA (8.73 g, 0.090 mol) and SO (8.80 g, assuming an average molecular weight of 880 g mol^−1^, 0.010 mol) were added to a 50 mL round-bottom flask and mixed thoroughly. The reaction system was purged with argon for approximately 10 min, after which the flask was immersed in an oil bath at 90 °C and allowed to react for 72 h. Upon completion, the mixture was cooled to room temperature, dissolved in THF, and washed with water to remove excess FA, glycerol, and traces of monoglycerides. The product was then lyophilized to yield SO–FA (conversion of ester groups: 88%; yield: 10.51 g, 60%). An additional wash with THF or HEX was performed to ensure complete removal of residual amine and ester.

**Fig. 1:**
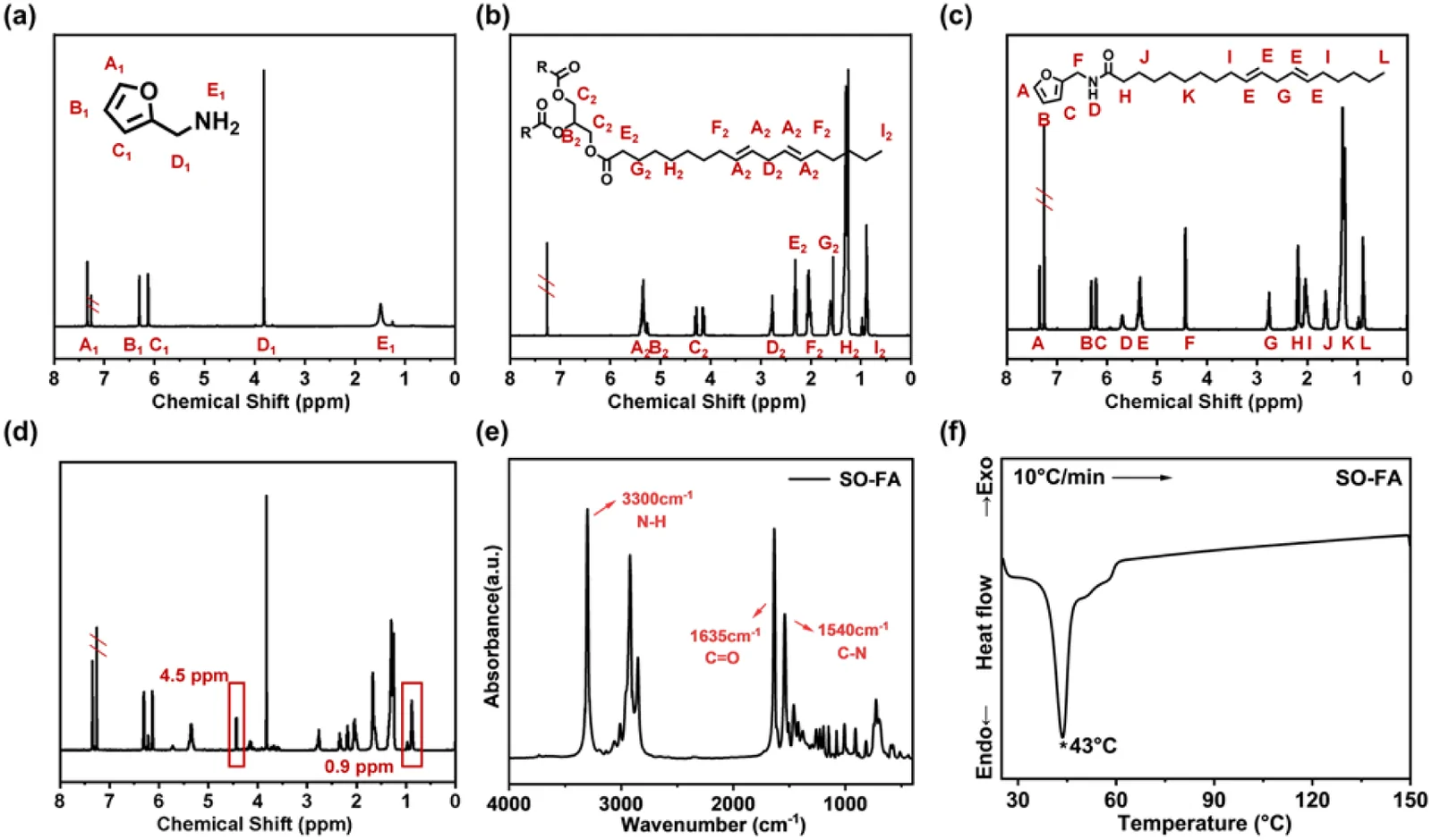
^1^H NMR spectra of (a) FA; (b) SO; (c) SO–FA; and (d) the SO/FA reaction system. (e) FTIR spectrum and (f) DSC curve of SO–FA.

Synthetic route (b) utilizes TBD as an organic base catalyst with a slight excess of amine, which ensures high atom economy while significantly shortening the reaction time and maintaining a high conversion rate of SO. In a typical procedure, FA (3.201 g, 0.033 mol), SO (8.80 g, 0.010 mol), and TBD (1.5 wt% relative to reactant) were charged into a 50 mL round-bottom flask and mixed thoroughly. The reaction system was purged with argon for approximately 10 min, after which the flask was immersed in an oil bath at 80 °C and allowed to react for 3 h. Upon completion, the mixture was cooled to room temperature, dissolved in THF, and washed with water to remove residual monomers and by-products. The product was then lyophilized to yield SO–FA (conversion of ester groups: 87%). Fig. 1 shows the ^1^H NMR spectrum of SO–FA, which was prepared *via* route (b). ^1^H NMR (400 MHz, chloroform-d) *δ*: 7.35 (d, *J* = 1.9 Hz, 1H), 6.32 (dd, *J* = 3.3, 1.9 Hz, 1H), 6.22 (d, *J* = 3.2 Hz, 1H), 5.70 (s, 1H), 5.44–5.27 (m, 3H), 4.44 (d, *J* = 5.3 Hz, 2H), 2.77 (t, *J* = 6.4 Hz, 1H), 2.19 (t, *J* = 7.6 Hz, 2H), 2.12–1.93 (m, 4H), 1.64 (q, *J* = 7.1 Hz, 2H), 1.28 (dt, *J* = 17.0, 5.5 Hz, 19H), 0.88 (dt, *J* = 7.0, 3.3 Hz, 3H).

#### 2.2.2. Synthesis of PO–FA

For the synthesis of PO–FA, PO (8.08 g, 0.010 mol), FA (3.201 g, 0.033 mol), and TBD (1.5 wt% relative to reactant) were charged into a 50 mL round-bottom flask. Under identical conditions, the reaction afforded PO–FA monomer with an ester group conversion of 95%. Fig. S1 shows the ^1^H NMR spectrum of PO–FA. ^1^H NMR (400 MHz, chloroform-d) *δ*: 7.35 (d, *J* = 1.8 Hz, 1H), 6.32 (dd, *J* = 3.2, 1.9 Hz, 1H), 6.22 (d, *J* = 3.2 Hz, 1H), 5.74 (s, 1H), 5.39–5.28 (m, 1H), 4.43 (d, *J* = 5.4 Hz, 2H), 2.23–2.14 (m, 2H), 2.00 (q, *J* = 6.7 Hz, 2H), 1.64 (q, *J* = 7.2 Hz, 2H), 1.37–1.26 (m, 11H), 1.26–1.22 (m, 12H), 0.91–0.85 (m, 3H).

#### 2.2.3. Synthesis of TO–FA

For TO–FA monomer, TO (8.74 g, 0.010 mol), FA (3.201 g, 0.033 mol), and TBD (1.5 wt% relative to reactant) were charged into a 50 mL round-bottom flask. Under identical conditions, the reaction afforded TO–FA monomer with an ester group conversion of 90%. Fig. S2 shows the ^1^H NMR spectrum of TO–FA. ^1^H NMR (400 MHz, chloroform-d) *δ*: 6.42–6.02 (m, 6H), 5.78–5.65 (m, 2H), 5.43–5.28 (m, 2H), 4.43 (d, *J* = 5.5 Hz, 2H), 4.40 (s, 1H), 2.22–1.95 (m, 8H), 1.73 (s, 1H), 1.68–1.58 (m, 4H), 1.46–1.40 (m, 1H), 1.30 (dddd, *J* = 20.7, 12.1, 8.7, 4.9 Hz, 24H), 0.94–0.80 (m, 5H).

### 2.3. Solution polymerization of SO–FA and MAH

**Scheme 2:**
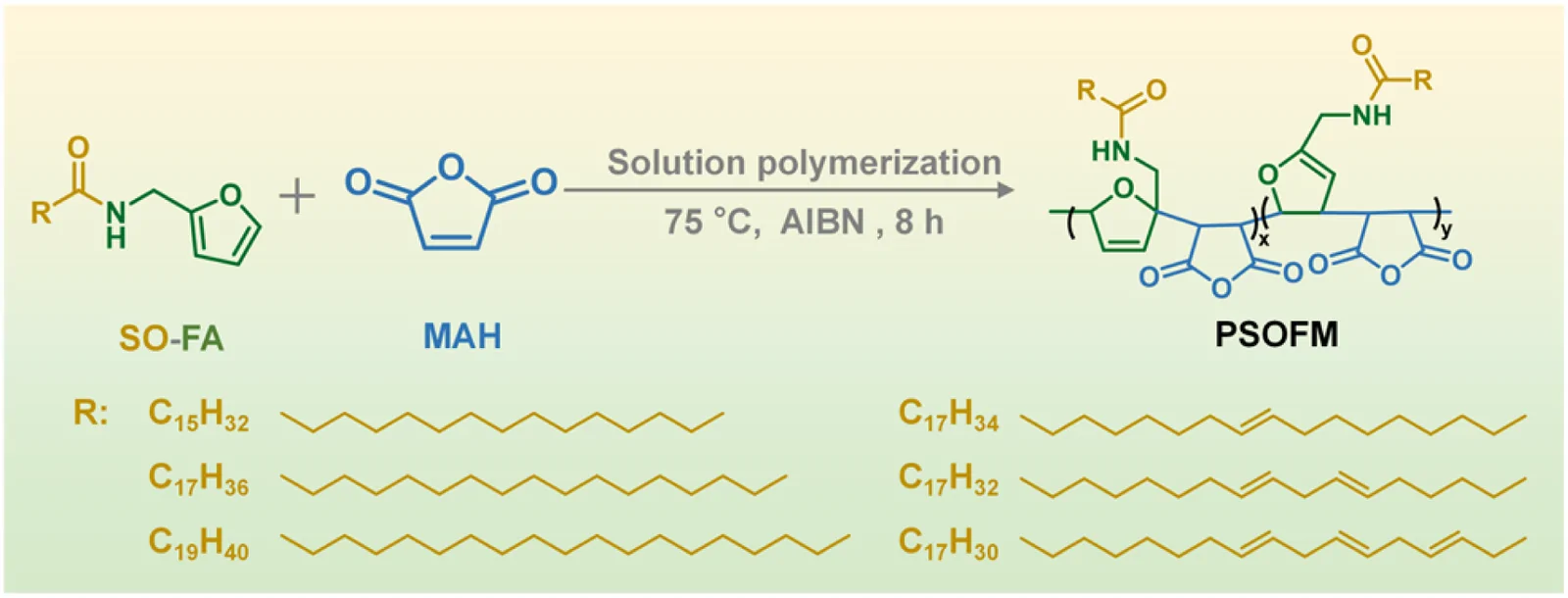
Synthesis of PSOFM.

As depicted in Scheme 2, in a typical procedure, SO–FA (10.00 g, 0.028 mol, assuming an average molecular weight of 360 g mol^−1^), MAH (2.72 g, 0.028 mol), and AIBN (0.636 g, 5 wt%) were dissolved in 58 mL (50.25 g) of IPA in a 100 mL round-bottom flask. The mixture was purged with argon for 20 min to remove dissolved oxygen, then immersed in an oil bath at 75 °C and stirred magnetically for 8 h. After the reaction, the product was precipitated, purified and dried to afford a predominantly alternating copolymer poly(SO–FA-*co*-MAH) (PSOFM, number average molecular weight (*M*_n_) = 9200 g mol^−1^, yield = 56%).

### 2.4. Measurement and characterization

Fourier transform infrared (FT-IR) spectra were recorded on a Nicolet Nexus 670 spectrometer over the wavenumber range of 400–4000 cm^−1^.

^1^H and ^13^C nuclear magnetic resonance (NMR) spectra were recorded on a Bruker Avance III 400 MHz, deuterated chloroform (CDCl_3_) was used as solvent for SO–FA, PO–FA and TO–FA monomers, while deuterated acetone (acetone-*d*_6_) was used for PSOFM.

The yield of PSOFM copolymer was measured by gravimetric method, and MAH conversion was measured by gas chromatography (GC) on a Shimadzu GC-2014.

The composition of PSOFM was determined *via* elemental analysis (C, H, O, N) performed on an Elementar Vario EL analyser (Germany), and the content of MAH units was calculated based on the experimental results.

The molecular weight of PSOFM copolymer was measured by Agilent 1260 infinity gel permeation chromatography (GPC) system equipped with two MIXED-C column and THF as the mobile phase at a flow rate of 1.0 mL min^−1^.

The polymer yield was determined by gravimetric method according to the following equation:
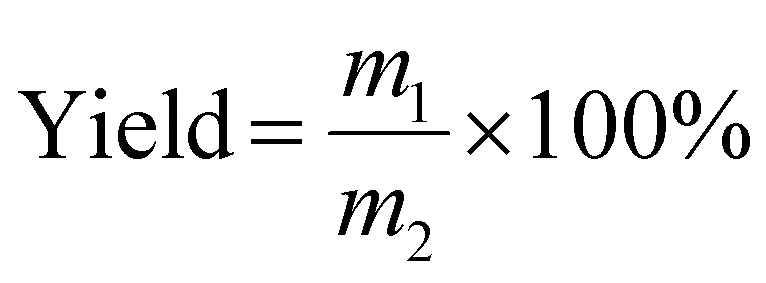
where *m*_1_ represents the mass of the obtained polymer and *m*_2_ represents the total mass of monomers before reaction.

The gel content was determined by Soxhlet extraction with THF for 36 h. Subsequently, the extracted residues were dried in a vacuum oven at 50 °C for 72 h. The gel content was calculated according to the following equation:
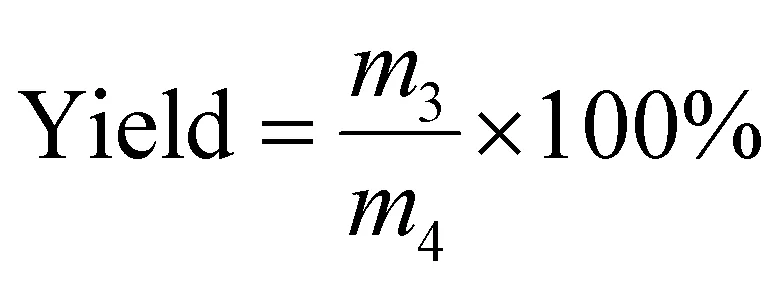
where *m*_3_ represents the mass of the polymer after extraction and *m*_4_ represents the initial mass of the polymer.

## 3. Results and discussion

### 3.1. Synthesis and characterization of SO–FA

The as-obtained VO–FA monomers were characterized by a series of analytical techniques (Fig. 1). For clarity, enlarged versions of these spectra are provided in the SI (Fig. S3). The ^1^H NMR spectra in Fig. 1a–d demonstrates that pure SO–FA monomer was obtained simply by dissolution in THF, followed by aqueous washing and sedimentation. The representative structure shown in the manuscript is drawn only for convenience of peak assignment and does not imply that the prepared SO–FA monomer is a single molecular species. As depicted in Fig. 1c, the signal of the furan proton in SO–FA at 6.11 ppm shifted to 6.22 ppm upon amide formation, whereas the signals at 6.32 ppm and 7.35 ppm remained unchanged.^30^ A new doublet peaks appeared at 4.44 ppm, corresponding to the newly formed –CH_2_– linking group between the amide and the furan ring, while the peaks at 4.16 ppm and 4.28 ppm of SO disappeared completely. Throughout the aminolysis process, the triplet signal at 0.88 ppm originated from the terminal –CH_3_ groups of SO fatty acid chains almost kept unchanged.^28,29^ Besides, other signals attributed to protons of fatty acid chains also remained almost unchanged in SO–FA monomer.

Additionally, SO–FA was further analysed *via* FTIR spectroscopy (Fig. 1e). Characteristic absorption bands for the amide group were observed at 1540 cm^−1^ (C–N), 1635 cm^−1^ (C=O), and 3300 cm^−1^ (N–H), respectively, while the peak at 1740 cm^−1^ ascribed to ester carbonyl C=O stretching vibration disappeared completely. Furthermore, the DSC curve shown in Fig. 1f indicates that the as-prepared SO–FA monomer undergoes a broad melting transition with a peak at approximately 43 °C. This phenomenon is mainly attributed to complex composition of the fatty acyl chains derived from SO, including differences in chain length and unsaturation degree, which lead to the formation of crystalline domains with different structural order and thermal stability. During heating process, the less ordered crystalline domains melt at lower temperature.^28^ To further verify this interpretation, single fatty acyl amide monomers derived from stearic acid, oleic acid, and linoleic acid were synthesized by reacting the corresponding fatty acids with furfurylamine (Fig. S4). DSC analysis of these model monomers and their mixture (Fig. S5) showed a thermal behaviour similar to that of SO–FA, confirming that the broad melting transition arises from the compositional heterogeneity of the soybean oil-derived fatty acyl chains.

Since the amine–ester exchange reaction is reversible and can reach an equilibrium state after sufficient reaction time, the reaction temperature is expected to influence both the chemical equilibrium shift and the reaction rate. To determine the conversion of SO to SO–FA, the reaction system was analysed by ^1^H NMR spectroscopy (Fig. 1d). As discussed above, the triplet signal at 0.9 ppm corresponding to terminal –CH_3_ groups of fatty acid chains almost remained unchanged during aminolysis process. Therefore, the amount of these terminal –CH_3_ groups kept constant in the reaction system, and the triplet signal at 0.9 ppm can serve as a reference. The conversion of ester groups in SO can be determined by the integral peak area ratio of the doublet peaks at 4.5 ppm (corresponding to the newly formed –CH_2_– linking group between the amide and the terminal –CH_3_ group of fatty acid chain).

#### 3.1.1. Influence of reaction temperature and amine-to-ester molar ratios on aminolysis of SO with FA

**Fig. 2:**
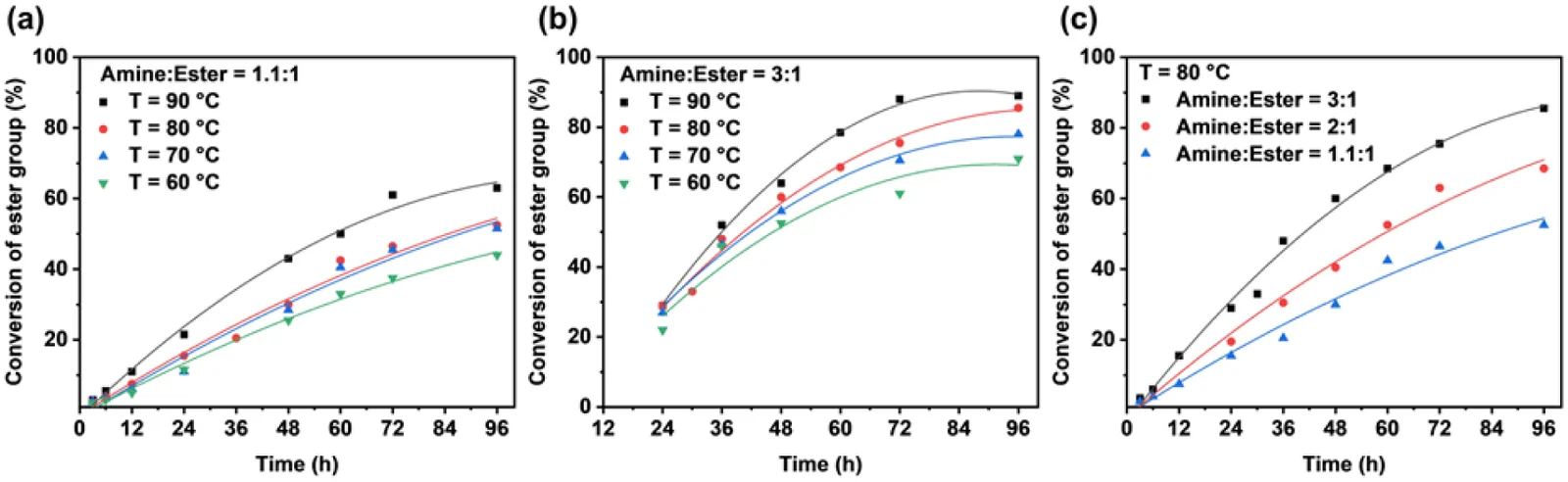
Variation of ester groups conversion *versus* reaction time: (a) at different temperatures with an amine-to-ester molar ratio of 1.1 : 1; (b) at different temperatures with an amine-to-ester molar ratio of 3 : 1; (c) at 80 °C with different amine-to-ester molar ratios.

As depicted in Fig. 2a and b, within the temperature range of 60–90 °C, elevated temperatures accelerated the rate of aminolysis reaction and enhanced conversion of ester groups. However, the overall reaction rate remained relatively low even at 90 °C, and the aminolysis reaction required approximately 72 h to approach equilibrium. It is worth pointing out that a higher amine-to-ester molar ratio led to an increase in final ester group conversion. After 72 h, the conversion of ester groups reached 61% and 88% at amine-to-ester molar ratios of 1.1 : 1 and 3 : 1, respectively. Fig. 2c further demonstrates that, at a fixed temperature, a markedly enhanced conversion rate of ester groups was achieved when the amine-to-ester molar ratio gradually increased. Notably, the influence of the amine-to-ester molar ratio on conversion of ester groups was more substantial than that of reaction temperature.

#### 3.1.2. Influence of catalysts on aminolysis of SO with FA

To investigate the influence of catalysts on aminolysis of SO with FA, different organic base catalysts were added into the reaction system. As shown in Fig. S6a, different catalysts exhibit varying effects on the amine–ester exchange reaction at 60 °C. Among them, TBD demonstrates the best catalytic performance, making it suitable for rapid synthesis of SO–FA monomer while meeting requirements of atom economy. Furthermore, Fig. S6b revealed that under the conditions of 1.5 wt% dosage of TBD at 80 °C for 3 hours, the conversion of ester groups decreased slightly with increasing amine-ester molar ratio, though it all remained above 80%.

With the addition of 1.5 wt% TBD, the conversion of ester groups reached 49% in 3 hours even at room temperature. It should be noted that the reaction system predominantly exists in a solid state at room temperature, severely hindering contact between unreacted reagents. Upon heating, the reaction system melts and changes from a solid to a liquid state at around 60 °C. Therefore, it is recommended to conduct the aminolysis reaction of SO at temperatures above 60 °C.

**Fig. 3:**
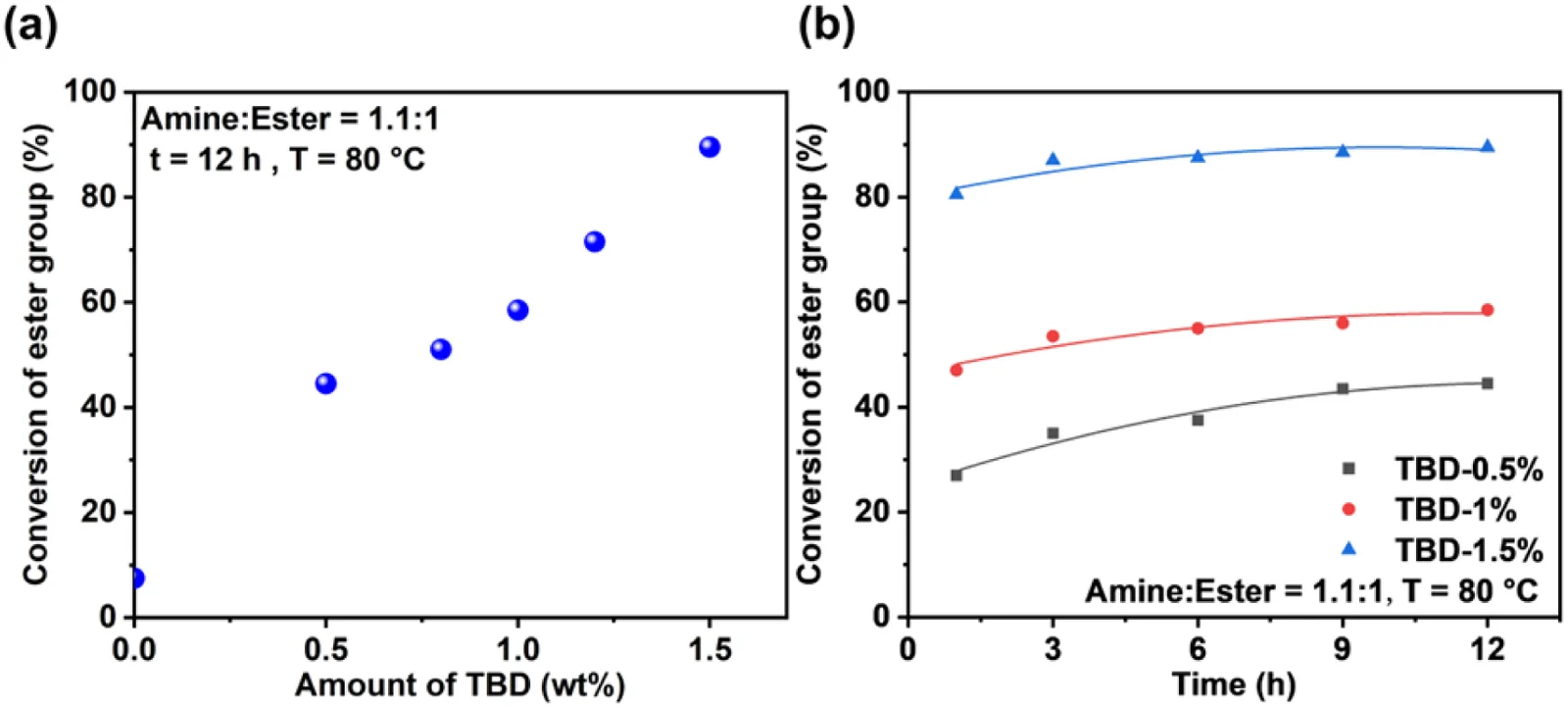
The influence of (a) dosage of TBD and (b) reaction time on conversion of ester groups. Reaction conditions: [amine] : [ester] = 1.1 : 1, at 80 °C for 12 h.

To determine the dosage of catalyst, the effect of TBD concentration on the aminolysis reaction of SO was examined in detail. As shown in Fig. 3a, at 80 °C for 12 hours, higher TBD dosage correlates with increased conversion of ester groups to amide groups. Combining this result with the kinetic curves shown in Fig. 3b, it can be clearly inferred that TBD can significantly enhance the aminolysis rate of SO with FA, especially during the early stage of reaction. At a dosage of 1.5 wt% TBD, the conversion of ester groups increased to 87% within the first 3 hours. Thereafter, the aminolysis degree (conversion of ester groups) increased marginally from 87% to 89.5% when the reaction time is extended from 3 h to 12 h. The rapid increase in ester groups conversion during the initial stage is mainly attributed to the bifunctional catalytic mechanism of TBD. Previous studies^31,32^ have demonstrated that TBD simultaneously activates both the amino nucleophile and the ester carbonyl group through hydrogen-bonding interactions, thereby accelerating the aminolysis reaction. Consequently, increasing the TBD loading provides more catalytic centres for substrate activation, enabling a larger fraction of ester groups to react within the same reaction time.

### 3.2. Solution copolymerization of SO–FA with MAH

#### 3.2.1. Characterization of PSOFM

**Fig. 4:**
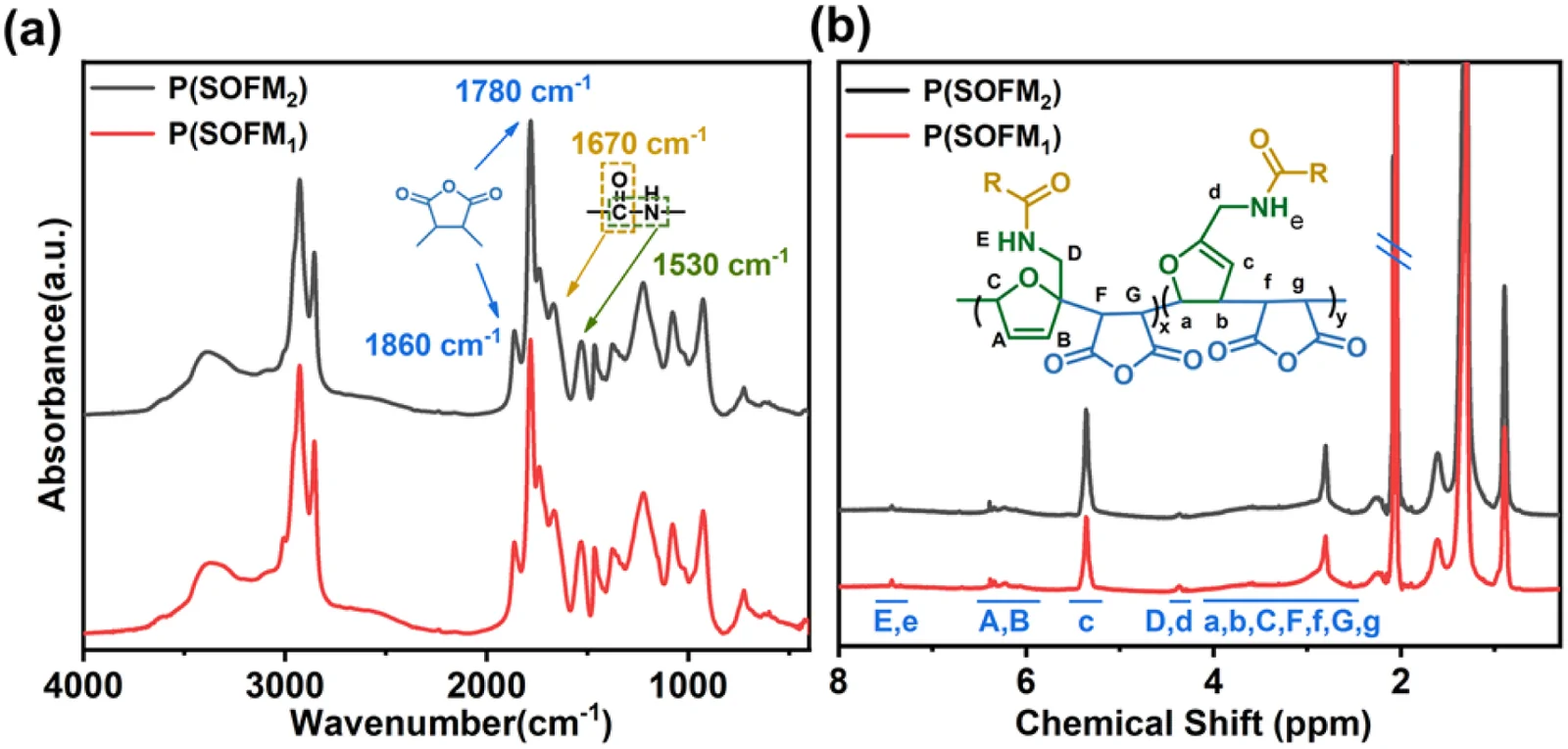
(a) FTIR spectra and (b) ^1^H NMR spectra of PSOFM.

Furan derivatives generally exhibit limited radical homopolymerization tendency, whereas their copolymerization with electron-deficient monomers has attracted considerable attention. Based on the special chemical structure and reactivity of SO–FA, MAH was employed as an electron-deficient comonomer to synthesize anhydride-functionalized copolymers. The copolymers prepared at MAH/SO–FA molar feed ratios of 2 : 1 and 1 : 1 were characterized by FTIR and ^1^H NMR spectroscopy. As shown in Fig. 4a, the as-obtained copolymer exhibits characteristic peaks at 1780 cm^−1^ and 1860 cm^−1^ corresponding to anhydride groups, as well as characteristic peaks at 1670 cm^−1^ and 1530 cm^−1^ attributed to amide group of SO–FA.

According to Fig. 4b, a broad peak appeared at 6.0–6.4 ppm corresponding to 1,4-addition polymer product of SO–FA, while a broad peak was observed at 3–4 ppm ascribed to the 1,2-addition polymer product. The ^1^H NMR spectrum confirmed that 1,2-addition polymer product predominated in the as-prepared PSOFM. The solid-state ^13^C NMR spectrum (Fig. S7) further confirms the presence of both 1,2- and 1,4-addition structures in the as-prepared PSOFM_1_. It is consistent with previous studies^33,34^ on the copolymerization of furan derivatives and maleic anhydride. Previous studies^33–36^ have shown that the donor–acceptor interaction between electron-rich furan derivatives and electron-deficient maleic anhydride favours alternating copolymerization. Therefore, it is expected that the copolymerization of SO–FA and maleic anhydride in the present work exhibits an alternating tendency. Owing to the current limitations of analytical techniques for structurally complex copolymers, a comprehensive characterization of the copolymer composition and sequence distribution remains challenging. Further studies are expected to provide a more complete understanding of the microstructure of the PSOFM copolymer.

The copolymer composition was further evaluated by elemental analysis and back-titration (Table S3). Elemental analysis revealed that the content of maleic anhydride units in the copolymers remained within the range of 36–39 wt% regardless of the initial monomer feed ratio, indicating an MAH/SO–FA molar ratio of approximately 1.5 : 1. Based on the results of copolymer composition, it can be speculated that the resulting PSOFM copolymer predominantly possesses an alternating structured backbone composed of MAH and SO–FA units, and there were also some MAH units attached to the fatty acyl side chains.^37^

Additionally, DSC analysis was performed to characterize the thermal properties of the VO-based copolymer, a glass transition temperature of approximately 63 °C was observed for the PSOFM_1_ copolymer according to Fig. S8.

#### 3.2.2. Influence of monomer molar ratios on copolymerization behaviour of MAH/SO–FA

**Fig. 5:**
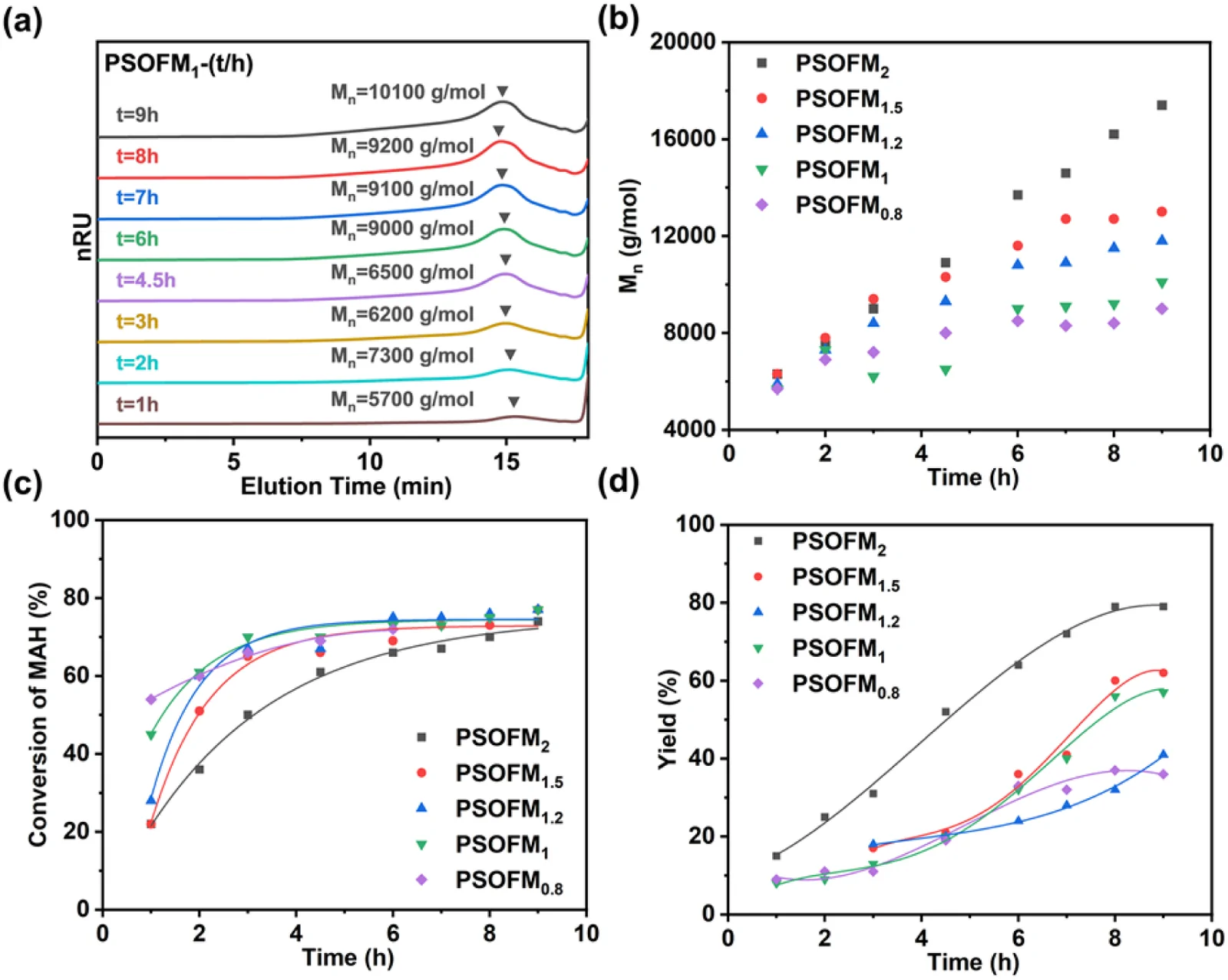
(a) The GPC curves of PSOFM_a_ (*a* = *n*(MAH)/*n*(SO–FA)) obtained at different reaction time. The plots of (b) *M*_n_; (c) MAH conversion; and (d) yield of PSOFM *versus* reaction time for copolymerization of MAH/SO–FA at different monomer molar ratios. Reaction condition: at 75 °C using 5 wt% AIBN with a monomer concentration of 20 wt%.

To investigate the influence of monomer molar ratios on copolymerization behaviour of MAH/SO–FA, MAH and SO–FA were dissolved in IPA at different molar feed ratios, with a fixed total monomers concentration of 20 wt% and an initiator dosage of 5 wt% relative to total monomers. The copolymerization was conducted at 75 °C for 9 h. The conversion of MAH, yield, *M*_n_, and polydispersity index (PDI) of PSOFM were determined by GC and GPC measurement. The standard curve and analytical method for MAH are provided in Fig. S9. The corresponding experimental results are provided in Table S2, Fig. 5, S10 and S11.

According to Fig. 5a, b, S10 and S11, the GPC curves for PSOFM obtained at different monomer feed ratios gradually shifted toward higher molecular weight direction with increasing reaction time, indicating a continuous increase in molecular weight. Meanwhile, a noticeable shoulder progressively appeared in the high molecular weight region. This phenomenon became more pronounced at higher MAH/SO–FA feed ratios, suggesting the formation of high-molecular-weight copolymers. The formation of high-molecular-weight PSOFM can be mainly attributed to the presence of unsaturated double bonds in the fatty acyl chain of SO–FA. Since the unsaturated double bonds in the fatty acyl chain also possess certain radical copolymerization reactivity toward MAH, branching polymerization and intermolecular coupling reactions may occur during copolymerization, thereby leading to the formation of high-molecular-weight PSOFM copolymer with broad molecular weight distribution.

According to Fig. 5c, the GC results showed that the content of MAH decreased rapidly within the initial 2 h, indicating that MAH was rapidly consumed through both Diels–Alder (D–A) reaction and radical copolymerization with SO–FA. After the initial stage, the concentration of MAH changed slightly, whereas the molecular weight of the resulting PSOFM copolymers continued to increase and meanwhile the molecular weight distribution became much broader. This result suggested that the D–A adducts formed in the early stage decomposed gradually and subsequently the regenerated MAH/SO–FA participated in copolymerization and branching reactions. As shown in Fig. 5d, the yields of PSOFM copolymers gradually increased with reaction time and approached a plateau at approximately 8 h. It is worthy to point out that higher MAH feed ratios resulted in higher PSOFM copolymer yields. Specifically, when the molar ratio of [MAH]/[SO–FA] increased from 1 : 1 to 2 : 1, the polymer yield increased from 56% to 79%. This behaviour can be attributed to the fact that an increased concentration of MAH can effectively enhanced the free radical copolymerization with SO–FA (furan ring and unsaturated double bonds in the fatty acyl chain), thereby remarkably promoting the incorporation of more SO–FA units into the copolymer product. Although higher polymer yield could be achieved at higher [MAH]/[SO–FA] feed ratios, the as-prepared PSOFM showed substantially broader molecular weight distributions, indicative of the formation of copolymer with highly branched molecular structure. Considering the trade-off between polymer yield and molecular structure, a feed ratio of [MAH]/[SO–FA] = 1/1 was selected for subsequent investigations of other reaction parameters.

#### 3.2.3. Influence of reaction conditions on copolymerization of SO–FA with MAH

**Fig. 6:**
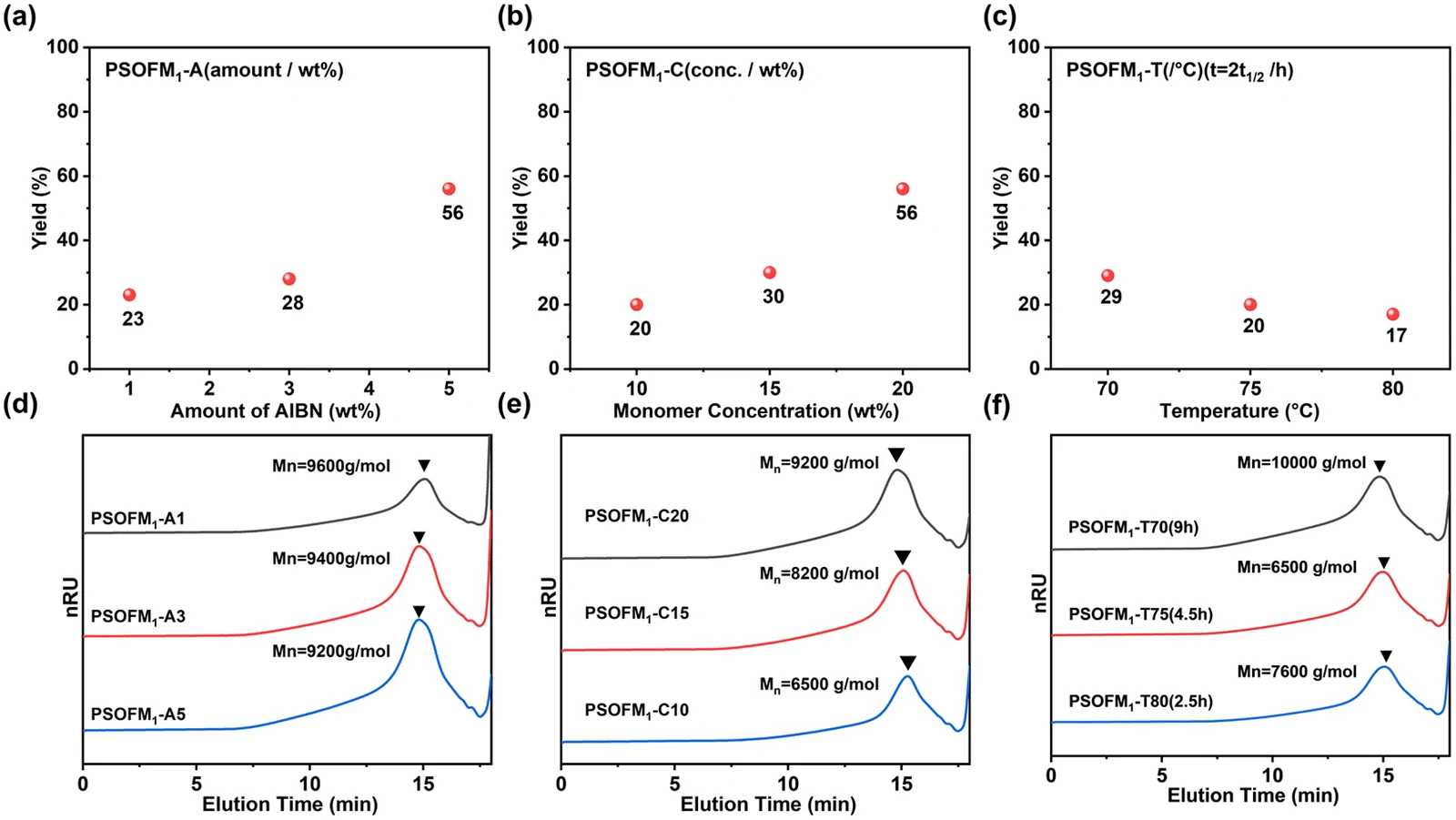
Plots of PSOFM copolymer yield as a function of (a) initiator dosage, (b) monomer concentration, and (c) temperature. (d)–(f) GPC traces of the corresponding PSOFM copolymers. Reaction conditions: [MAH]/[SO–FA] = 1/1; for PSOFM_1_-A, at 75 °C for 8 h with fixed total monomers concentration of 20 wt%; for PSOFM_1_-C, at 75 °C for 8 h using 5 wt% AIBN relative to total monomers; for PSOFM_1_-T, 5 wt% AIBN and a total monomers concentration of 20 wt% were employed.

The influence of reaction conditions on copolymerization of MAH/SO–FA was investigated systematically, and the detailed formulations of the copolymerization system are summarized in Table S3. For free-radical polymerization, the initiator concentration plays a crucial role in determining both the polymerization rate, monomer conversion and molecular weight of the resulting polymer. Therefore, the copolymerization behaviour of SO–FA and MAH under different AIBN concentrations was investigated in detail. Using isoamyl acetate as solvent, the copolymerization was carried out under an equimolar SO–FA/MAH feed ratio at 75 °C. The polymer yields and GPC curves of the corresponding PSOFM are presented in Fig. 6a and d, and the experimental results are summarized in Table S3a. The experimental results listed in Table S3a demonstrate that the azo initiator AIBN exhibited significantly higher initiation efficiency than peroxide initiators. This can be attributed to the relatively low decomposition temperature and efficient radical generation capability of AIBN, which facilitated the formation of monomer radicals (propagating radical species) and promoted copolymerization between SO–FA and MAH. In contrast, peroxide initiators showed relatively lower initiation efficiency under the same reaction conditions, resulting in lower monomer conversion.

As the AIBN dosage increased from 1 wt% to 5 wt%, the total monomers conversion gradually increased from 23% to 56%. The enhanced monomer conversion is mainly associated with the higher concentration of primary radicals generated at elevated initiator dosages, which effectively accelerated radical chain initiation and propagation processes, thereby facilitating the formation of PSOFM copolymer. However, excessive initiator may also greatly accelerate the chain termination reactions and lead to the formation of polymers with relatively low molecular weights. The experimental results are consistent with that of conventional radical polymerization process. Considering both monomer conversion and molecular weight of the as-prepared PSOFM copolymer, the maximum AIBN concentration was set to be 5 wt%.

Besides initiator type and dosage, monomer concentration may also significantly affect the copolymerization behaviour. Therefore, SO–FA and MAH were copolymerized at an equimolar feed ratio under different total monomer concentrations to investigate the influence of monomer concentration on copolymerization. When the concentration of SO–FA exceeded 20 wt%, SO–FA could not completely dissolve in isoamyl acetate at room temperature. Therefore, the monomer concentration range was limited to 10–20 wt%. As shown in Fig. 6b and e, and Table S3b, with increasing monomer concentrations, higher monomer conversion was achieved, accompanied by a left-shift of the GPC curves, indicating the formation of PSOFM copolymers with higher molecular weight. According to the experimental results, the highest polymer yield of 56% (*M*_n_ = 9200 g mol^−1^, PDI = 23.7) was obtained when the copolymerization was conducted at 75 °C for 8 h with 20 wt% total monomers concentration and 5 wt% initiator relative to total monomers. Notably, the as-prepared PSOFM possessed an extremely broad molecular weight distribution, which was probably due to the participation of the unsaturated double bonds in the fatty acyl chain of SO–FA in copolymerization.

Under the optimized reaction conditions described above, the effect of reaction temperature on the copolymerization behaviour of MAH/SO–FA was further investigated. The polymerization time was set to be twice the half-life of the initiator, and the experimental data are shown in Fig. 6c and f, and Table S3c. It is obvious from Fig. 6c and f that, with increasing reaction temperature, the polymer yield decreased gradually and meanwhile *M*_n_ values for the as-obtained PSOFM became lower. Moreover, the molecular weight distribution became narrower. The variation of polymer yield is totally different from that of conventional radical polymerization process, which increased gradually with increasing temperature. This phenomenon can be mainly attributed to the remarkably accelerated Diels–Alder reaction between MAH and SO–FA at elevated temperatures, which generated high concentration of Diels–Alder product without radical polymerization activity within a short period. Therefore, the polymer yield decreased at higher polymerization temperature. Furthermore, higher reaction temperatures may greatly promote chain termination reactions, thereby suppressing effective chain propagation. Consequently, PSOFM copolymers with relatively lower molecular weights and narrower molecular weight distributions were obtained at higher temperatures.

#### 3.2.4. Mechanism for copolymerization of SO–FA with MAH

As discussed above, the participation of unsaturated double bonds in the fatty acyl chain of SO–FA in radical copolymerization with MAH was proposed to elucidate the formation of PSOFM copolymers with extremely high molecular weight. To further confirm the possible involvement of double bonds in the fatty acyl chain during copolymerization, the radical copolymerization of the single fatty acyl amide monomers with MAH was carried out in isoamyl acetate under an equimolar feed ratio of single fatty acyl amide/MAH at 75 °C. The synthesized copolymers are designated as PSFM, POFM and PLFM, respectively.

After copolymerization with MAH, obvious precipitation was observed for the reaction systems based on oleamide and linoleamide monomers, whereas the stearamide monomer-based reaction system still remained completely soluble. Gravimetric method and gel content measurements demonstrated that the copolymer yields were 21% for PSFM, 66% for POFM, and 68% for PLFM. Furthermore, POFM and PLFM exhibited a gel content of 45% and 63%, respectively. These observations suggest that the unsaturated double bonds in the fatty acyl chains can effectively participate in the radical copolymerization with MAH. When the unsaturation degree of VO–FA monomers is sufficiently high, copolymers with branched or even crosslinked structure will be formed.

**Fig. 7:**
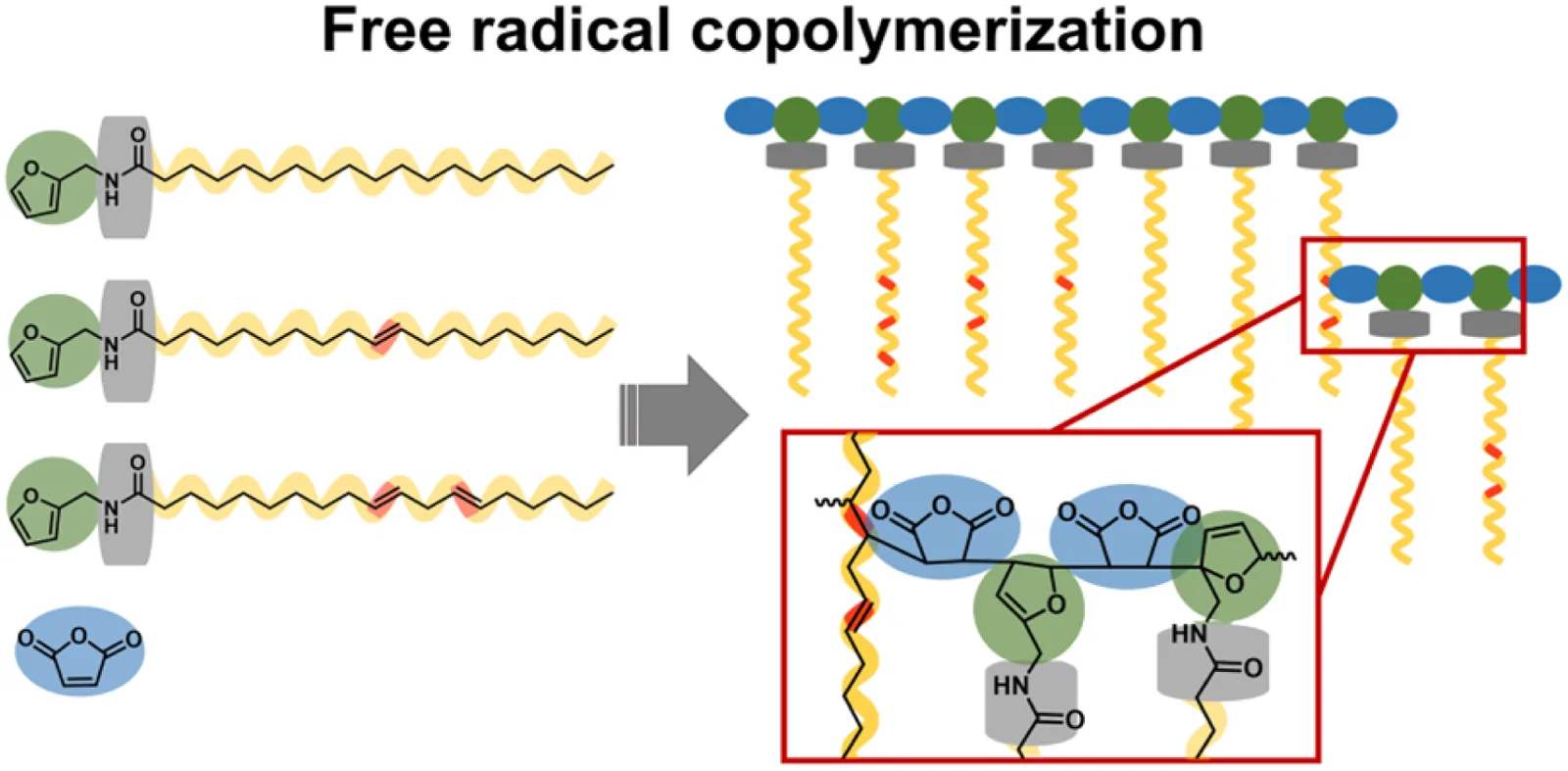
A possible mechanism for copolymerization of SO–FA with MAH.

GPC analysis was further performed for the supernatants of the three reaction systems. As depicted in Fig. S12 and Table S4, the experimental results showed that the GPC curves of the as-prepared copolymers gradually shifted toward the high-molecular-weight region with increasing content of double-bond in the fatty acyl chain, indicating the formation of copolymers with higher molecular weights. This phenomenon could be explained based on the additional reactive sites provided by the unsaturated double bonds in the fatty acyl chain, which may facilitate not only branching polymerization but also intermolecular chain coupling reaction during copolymerization process. Based on the experimental results and the reaction mechanisms of MAH with carbon–carbon double bonds reported in previous studies,^37–39^ a possible mechanism for copolymerization of SO–FA with MAH was proposed in Fig. 7. During the copolymerization process, SO–FA containing unsaturated double bonds in the fatty acyl chain could serve as multifunctional monomers to perform branching polymerization, thereby resulting in copolymer with higher molecular weight and broader molecular weight distribution.

## 4. Conclusions

In this work, a series of VO–FA monomers, including SO–FA, PO–FA, and TO–FA, were successfully synthesized through a solvent-free aminolysis reaction between VO and FA. Using SO as a model substrate, the uncatalyzed reaction reached equilibrium after 72 h at 90 °C, affording an ester conversion of 61% at an amine-to-ester ratio of 1.1 : 1. The introduction of organic base catalysts markedly accelerated the aminolysis process, and TBD exhibited the highest catalytic activity, achieving 87% ester conversion within only 3 h at 80 °C with 1.5 wt% catalyst loading. Taking advantage of the furan group incorporated in SO–FA, a novel fully bio-based copolymer (PSOFM) was successfully prepared *via* radical copolymerization with MAH. PSOFM was obtained with a yield of 56% and *M*_n_ of 9200 g mol^−1^ under the optimized conditions of an equimolar SO–FA/MAH feed ratio, 5 wt% AIBN, and 20 wt% monomer concentration at 75 °C for 8 h. Structural analyses revealed that the unsaturated double bonds in the fatty acid side chains of SO–FA participated in the copolymerization process, resulting in the formation of branched copolymer. Higher side-chain unsaturation facilitated branching polymerization, leading to the formation of PSOFM with increased molecular weight and broader molecular weight distribution. Owing to their fully bio-based nature, flexible aliphatic side chains, and abundant reactive anhydride groups, these VO-based polymers show considerable promise as sustainable toughening agents and advanced functional polymer materials.

## Author contributions

Jundan Chen: data curation, investigation, writing – original draft, visualization and formal analysis. Yanbei Chen: visualization. Xianhong Zhang: conceptualization, methodology. Dong Chen: conceptualization, methodology, validation, writing – review and editing. Yuhong Ma: conceptualization, methodology. Wantai Yang: supervision, funding acquisition.

## Conflicts of interest

There are no conflicts to declare.

## Supplementary Material

RA-OLF-D6RA05111A-s001

## Data Availability

All data generated or analyzed during this study are included in this published article and its supplementary information (SI). Supplementary information: NMR, GC, FTIR, DSC, and GPC results, and the detailed synthetic procedures of single fatty acyl amide monomers. See DOI: https://doi.org/10.1039/d6ra05111a.
