# Supplementary material for: Synthesis of vegetable oil-based furfurylamine-derived monomer and its polymerization behaviour
Source: RSC Adv. 2026 Aug 25;16(42):50332–40. doi: 10.1039/d6ra05111a (PMC13504664; doi:10.1039/d6ra05111a)
Supplement: Supplementary file 1 [file RA-016-D6RA05111A-s001.pdf]

Supplementary Information (SI) for RSC Advances.
This journal is © The Royal Society of Chemistry 2026

Supporting Information

Synthesis of vegetable oil-based furfurylamine-derived monomer and its polymerization behaviour

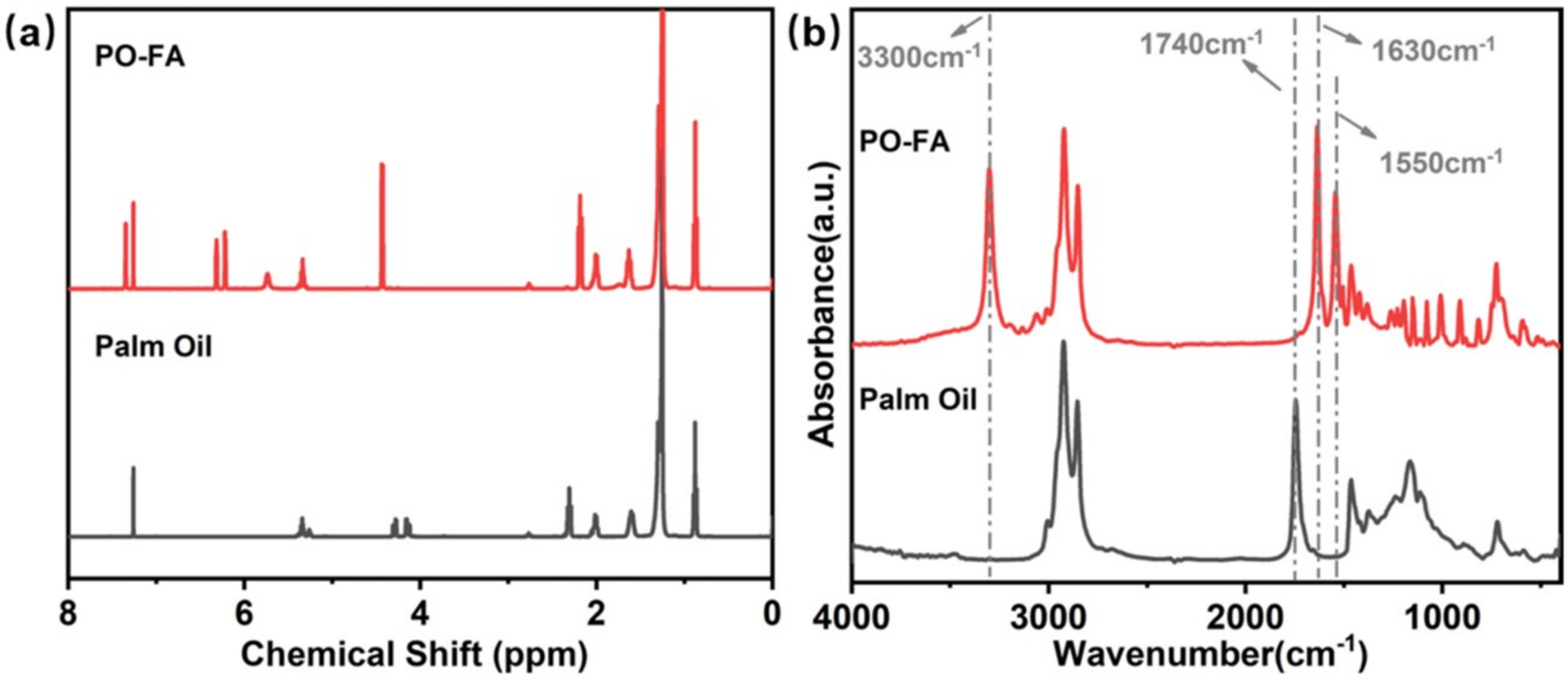

Figure S1. (a) ^{1}H NMR spectra and (b) FTIR spectra of PO-FA and PO.

PO-FA: ^{1}H NMR (400 MHz, Chloroform-d)δ 7.35 (d, J = 1.8 Hz, 1H), 6.32 (dd, J = 3.2, 1.9 Hz, 1H), 6.22 (d, J = 3.2 Hz, 1H), 5.74 (s, 1H), 5.39 - 5.28 (m, 1H), 4.43 (d, J = 5.4 Hz, 2H), 2.23 - 2.14 (m, 2H), 2.00 (q, J = 6.7 Hz, 2H), 1.64 (q, J = 7.2 Hz, 2H), 1.37 - 1.26 (m, 11H), 1.26 - 1.22 (m, 12H), 0.91 - 0.85 (m, 3H).

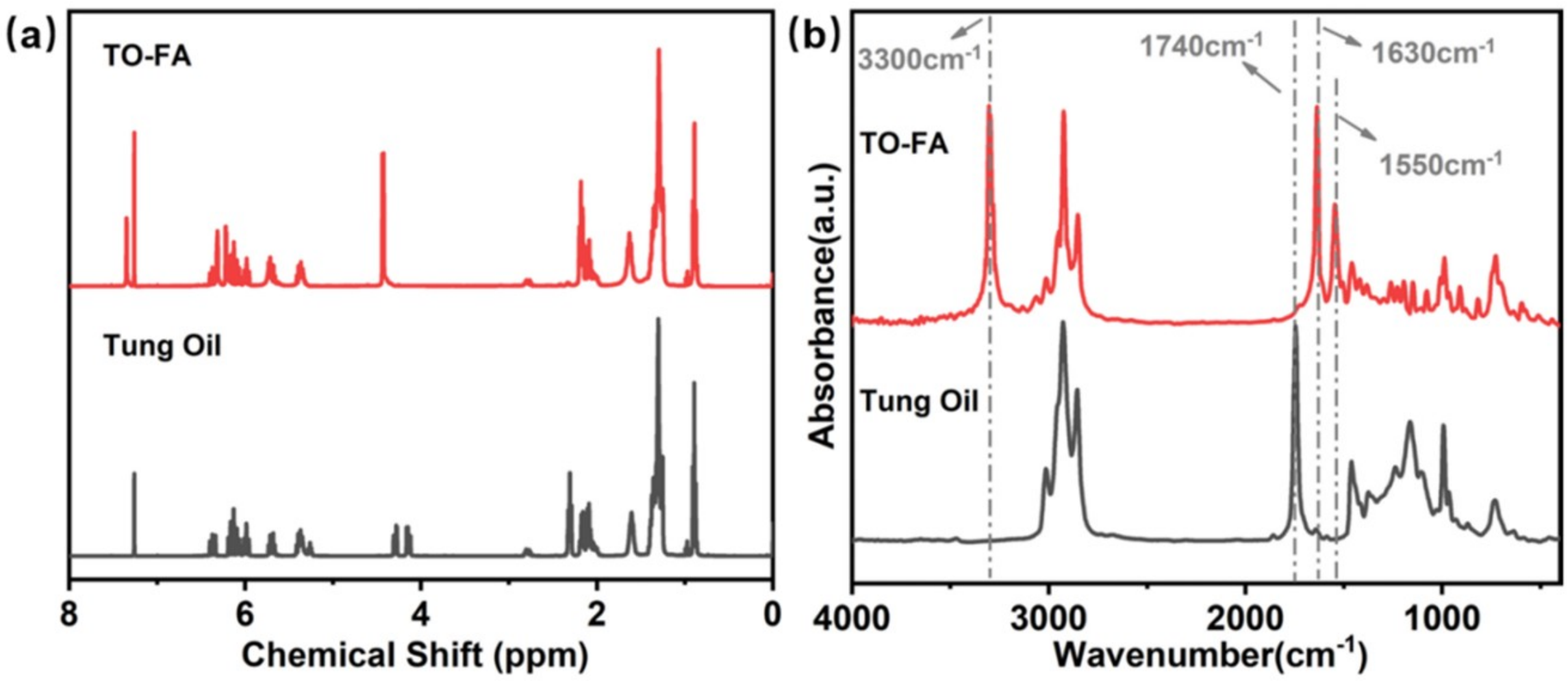

Figure S2. (a) ^{1}H NMR spectra and (b) FTIR spectra of TO-FA.

TO-FA: ^{1}H NMR (400 MHz, Chloroform-d) δ 6.42 - 6.02 (m, 6H), 5.78 - 5.65 (m, 2H), 5.43 - 5.28 (m, 2H), 4.43 (d, J = 5.5 Hz, 2H), 4.40 (s, 1H), 2.22 - 1.95 (m, 8H), 1.73 (s, 1H), 1.68 - 1.58 (m, 4H), 1.46 - 1.40 (m, 1H), 1.30 (dddd, J=20.7, 12.1, 8.7, 4.9 Hz, 24H), 0.94 - 0.80 (m, 5H).

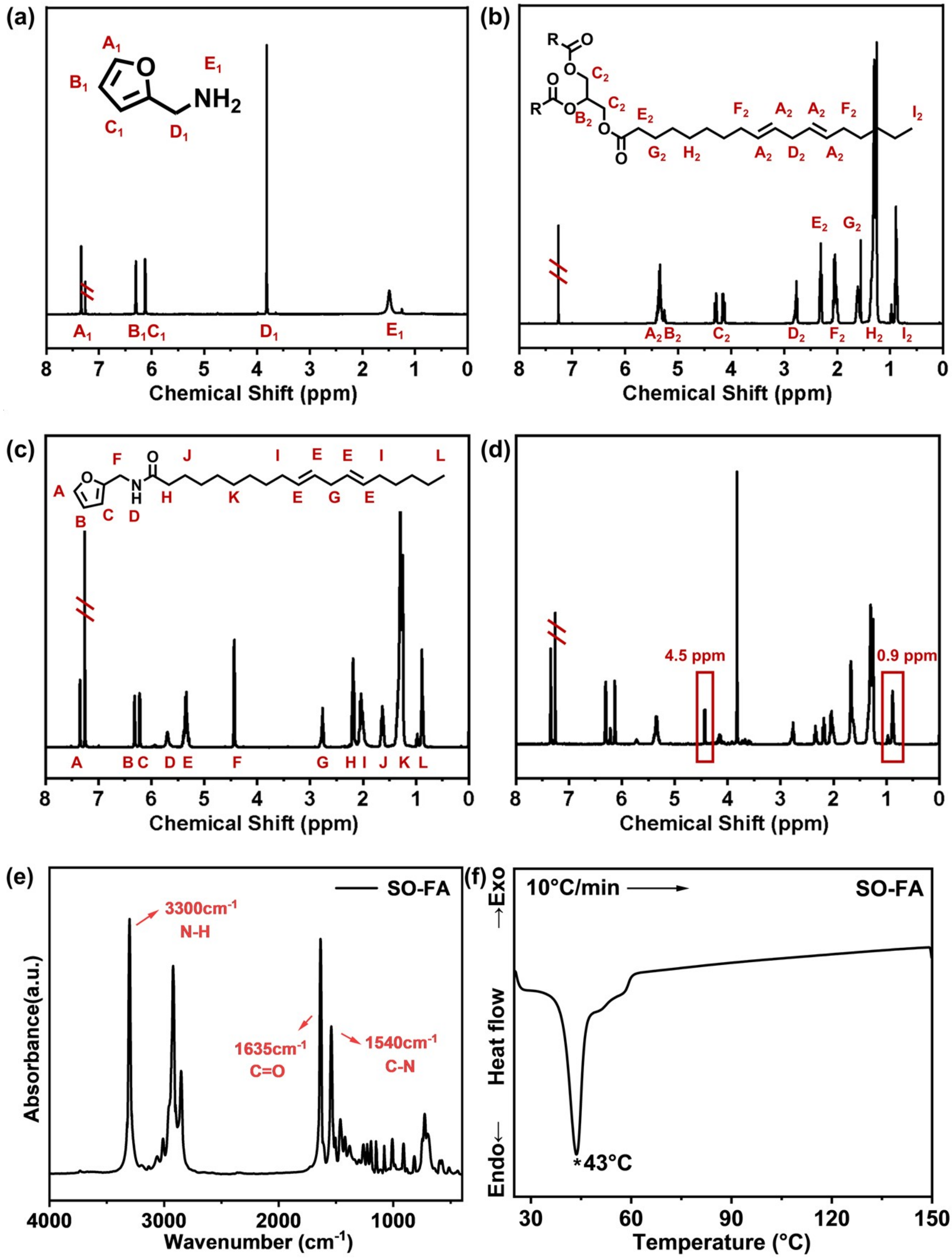

Figure S3. ^{1}H NMR spectra of (a) FA; (b) SO; (c) SO-FA; and (d) the SO/FA reaction system. (e) FTIR spectrum and (f) DSC curve of SO-FA.

FA: ^{1}H NMR (400 MHz, Chloroform-d) δ 7.34 (dd, J = 1.9, 0.9 Hz, 1H), 6.30 (dd, J = 3.2, 1.8 Hz, 1H), 6.12 (dd, J = 3.2, 0.9 Hz, 1H), 3.82 (d, J = 0.8 Hz, 2H), 1.63-1.38 (m, 2H).

SO: ^{1}H NMR (400 MHz, Chloroform-d) δ 5.44 – 5.30 (m, 4H), 5.34 – 5.21 (m, 1H), 4.29 (dd, J = 11.9, 4.3 Hz, 1H), 4.14 (dd, J = 11.9, 6.0 Hz, 1H), 2.79 (dt, J = 13.0, 6.3 Hz, 2H), 2.31 (td, J = 7.5, 2.6 Hz, 3H), 2.10 – 1.96 (m, 5H), 1.61 (tq, J = 10.4, 7.1, 5.6 Hz,

3H), 1.41-1.32 (m, 1H), 1.36 (s, 1H), 1.30 (q, J = 5.3 Hz, 15H), 1.27 (d, J = 3.5 Hz, 1H), 1.25 (s, 4H), 0.88 (td, J = 6.8, 3.9 Hz, 4H).

SO-FA: ^{1}H NMR (400 MHz, Chloroform-d) δ: 7.35 (d, J = 1.9 Hz, 1H), 6.32 (dd, J = 3.3, 1.9 Hz, 1H), 6.22 (d, J = 3.2 Hz, 1H), 5.70 (s, 1H), 5.44-5.27 (m, 3H), 4.44 (d, J = 5.3 Hz, 2H), 2.77 (t, J = 6.4 Hz, 1H), 2.19 (t, J = 7.6 Hz, 2H), 2.12-1.93 (m, 4H), 1.64 (q, J = 7.1 Hz, 2H), 1.28 (dt, J = 17.0, 5.5 Hz, 19H), 0.88 (dt, J = 7.0, 3.3 Hz, 3H).

Synthesis and Characterization of a Single-Component Fatty Acid Amide

Materials: Oleic acid (99%), linoleic acid (98%), and anhydrous tetrahydrofuran (THF (anhydrous) ,99.9%) were supplied by Energy Chemical. Stearic acid (>98%) was provided by Tokyo Chemical Industry Co., Ltd. 2-Furfurylamine (FA, 99%) and N,N'-carbonyldiimidazole (CDI, ≥99%) were obtained from Aladdin Industrial Corporation. Ethyl acetate (EAC) was supplied by Tianjin Fuchen Chemical Reagent Factory. Deionized water was prepared in our laboratory.

Synthesis: In a typical procedure, CDI (1.2 equiv, 19.46 g) and the single fatty acid (1 equiv: stearic acid, 28.45 g; oleic acid, 28.25 g; or linoleic acid, 28.04 g) were completely dissolved in THF (anhydrous) in a 250 mL three-necked flask. The reaction system was purged with argon, and the mixture was activated for 1 hour. Subsequently, FA (1.1 equiv, 10.68 g) was added dropwise via a dropping funnel. The reaction was then allowed to proceed at 40 °C for 12 hours. Upon completion, the reaction was quenched by adding water (approximately twice the volume of the reaction mixture). The mixture was extracted with ethyl acetate (3 times). The combined organic phases were washed with saturated brine, dried over anhydrous sodium sulfate, and filtered. The solvent was removed under reduced pressure to afford the crude product. The crude product was dissolved in THF and then precipitated in ice-water to yield the corresponding pure product. The synthesized amides will be designated as follows: stearic acid furfuryl amide as S-FA, oleic acid furfuryl amide as O-FA, and linoleic acid furfuryl amide as L-FA.

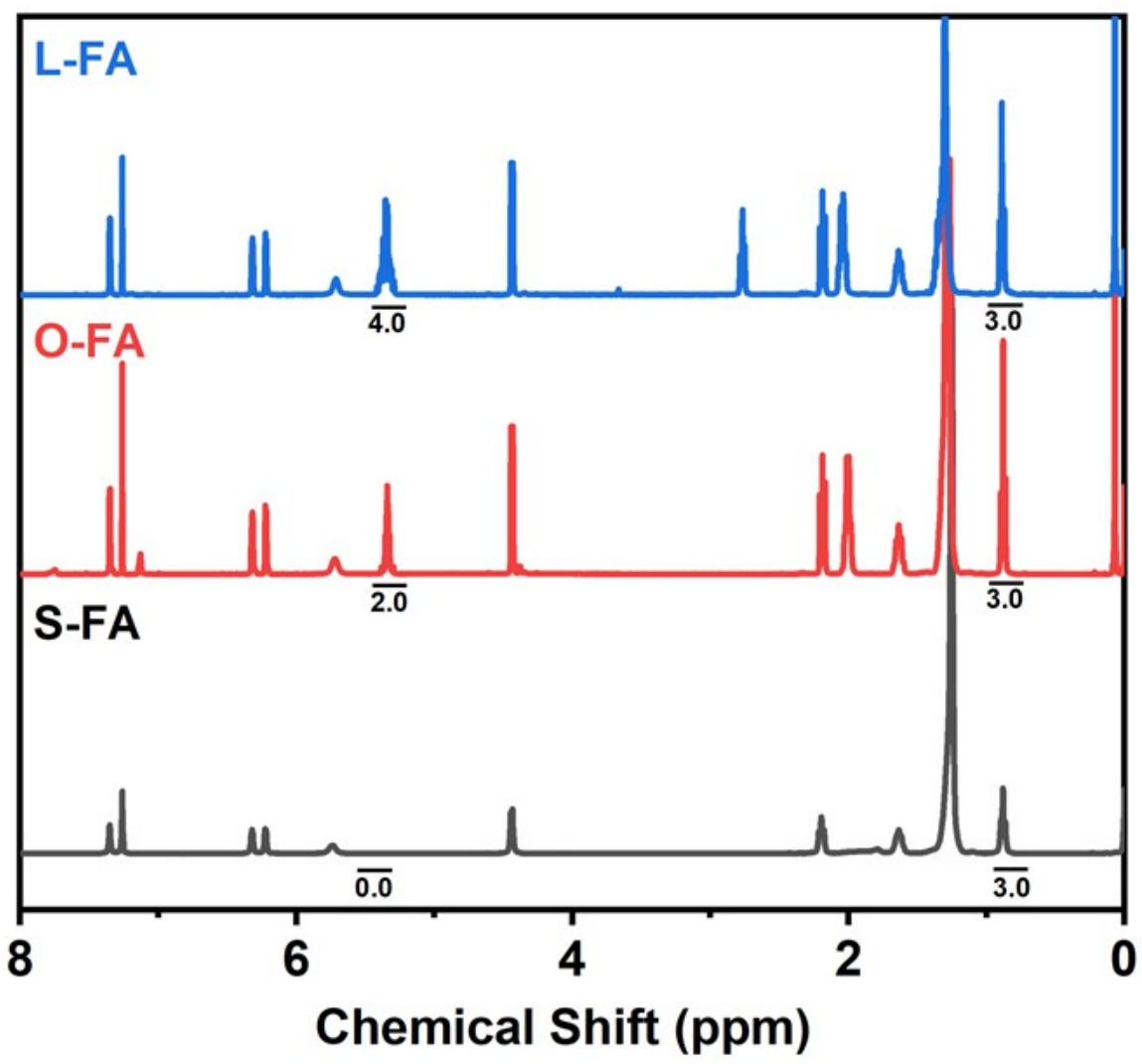

Figure S4. ^{1}H NMR spectra of S-FA, O-FA and L-FA.

S-FA: ^{1}H NMR (400 MHz, Chloroform-d). δ 7.39 - 7.32 (m, 1H), 6.36 - 6.29 (m, 1H), 6.22 (d, J = 3.3 Hz, 1H), 5.74 (s, 1H), 4.44 (d, J

= 5.0 Hz, 2H), 2.19 (t, J = 7.7 Hz, 2H), 1.63 (p, J = 7.1 Hz, 2H), 1.51 - 1.05 (m, 31H), 0.88 (t, J = 6.6 Hz, 3H).

O-FA: ^{1}H NMR (400 MHz, Chloroform-d). δ 7.35 (d, J = 1.9 Hz, 1H), 6.32 (t, J = 2.6 Hz, 1H), 6.22 (d, J = 3.2 Hz, 1H), 5.72 (s, 1H), 5.39 - 5.29 (m, 2H), 4.43 (d, J = 5.4 Hz, 2H), 2.19 (t, J = 7.6 Hz, 2H), 2.00 (q, J = 6.5 Hz, 4H), 1.64 (q, J = 7.3 Hz, 2H), 1.38 - 1.21 (m, 20H), 0.88 (t, J = 6.7 Hz, 3H).

L-FA: ^{1}H NMR (400 MHz, Chloroform-d). δ 7.35 (dd, J = 1.9, 0.9 Hz, 1H), 6.32 (dd, J = 3.2, 1.9 Hz, 1H), 6.22 (d, J = 3.2 Hz, 1H), 5.71 (s, 1H), 5.45 - 5.27 (m, 4H), 4.44 (d, J = 5.4 Hz, 2H), 2.77 (t, J = 6.4 Hz, 2H), 2.23 - 2.13 (m, 2H), 2.04 (q, J = 6.6, 2.3 Hz, 4H), 1.69 - 1.57 (m, 2H), 1.42 - 1.22 (m, 14H), 0.89 (t, J = 6.8 Hz, 3H).

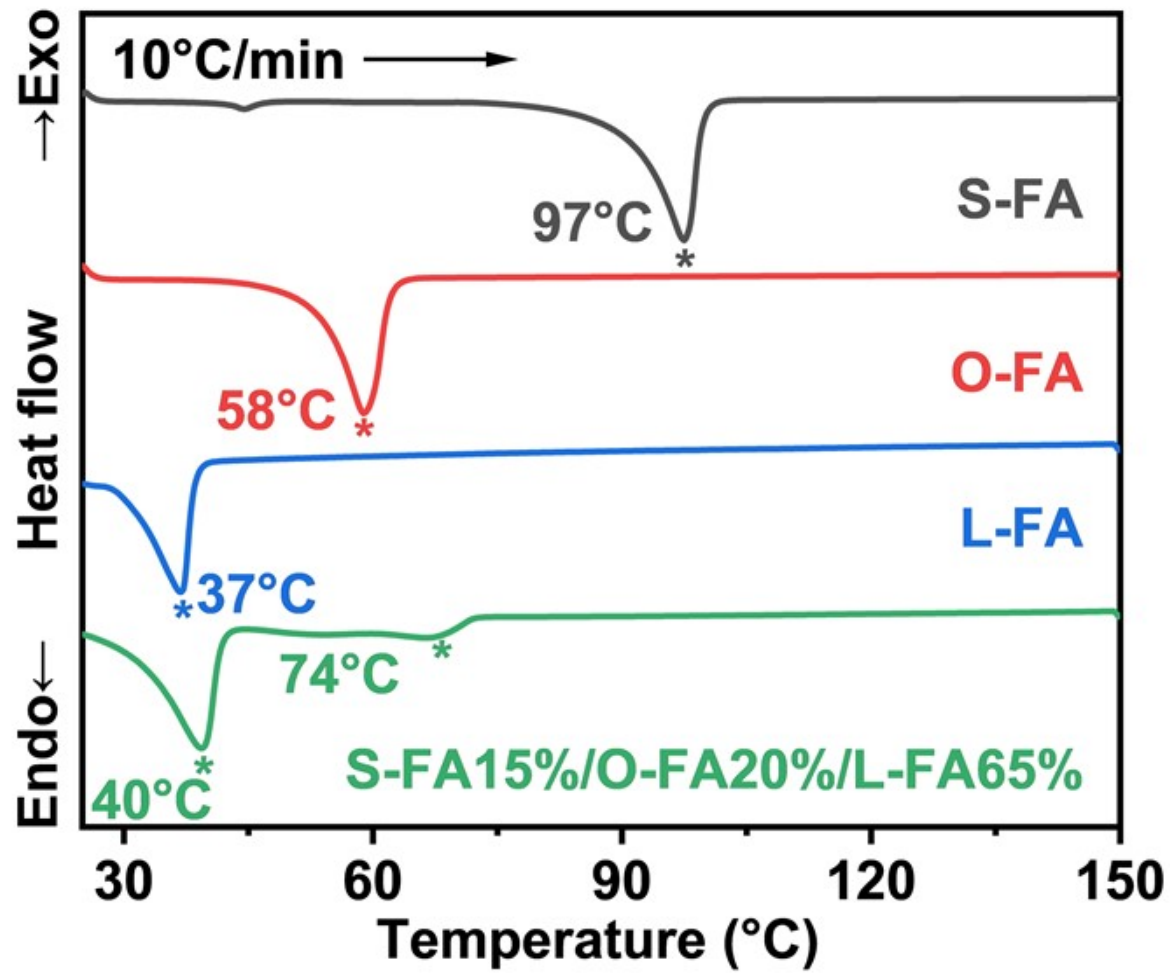

Figure S5. DSC curves of S-FA, O-FA, L-FA and mixture of S-FA15%/O-FA20%/L-FA65%.

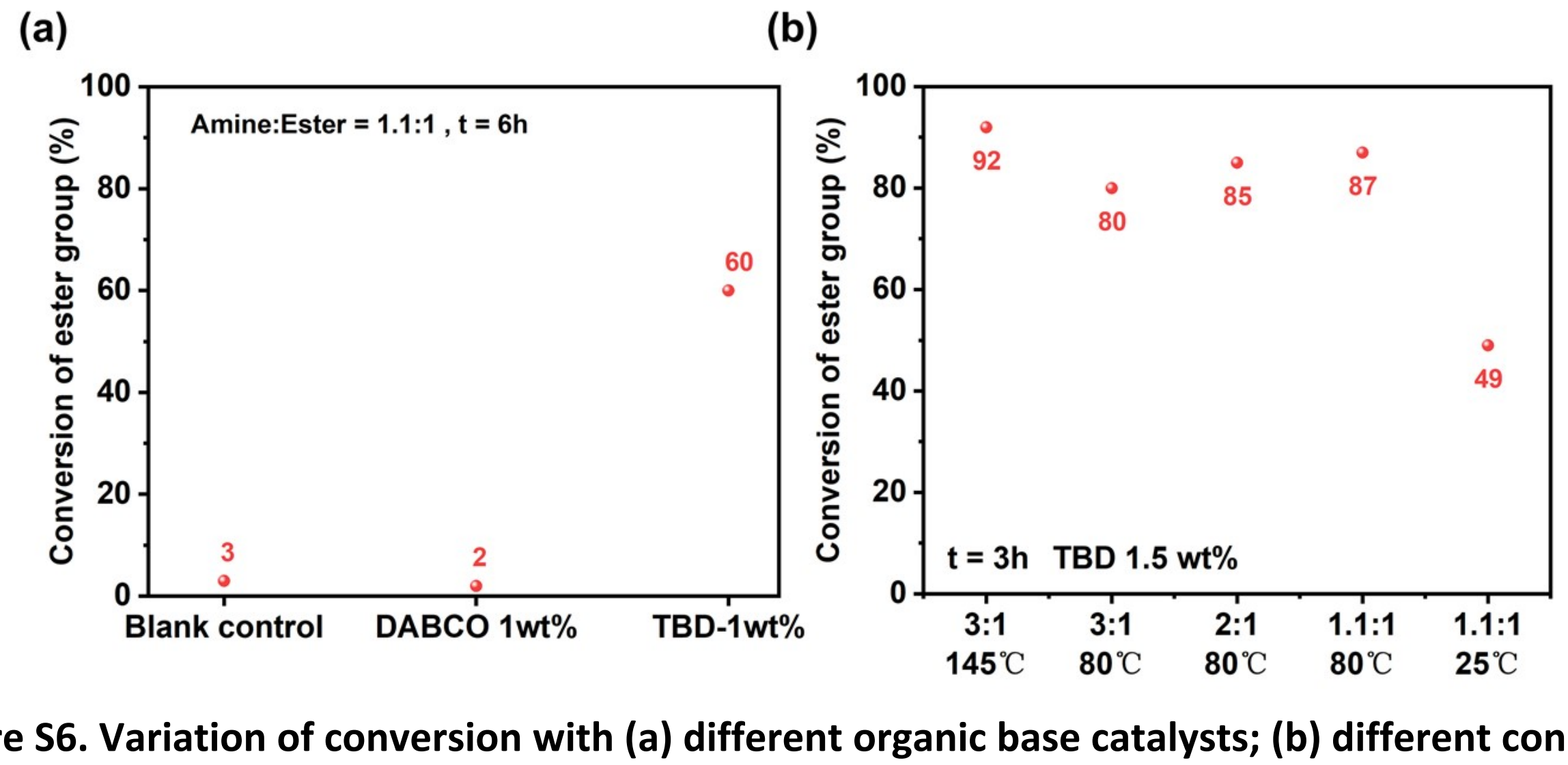

Figure S6. Variation of conversion with (a) different organic base catalysts; (b) different condition.

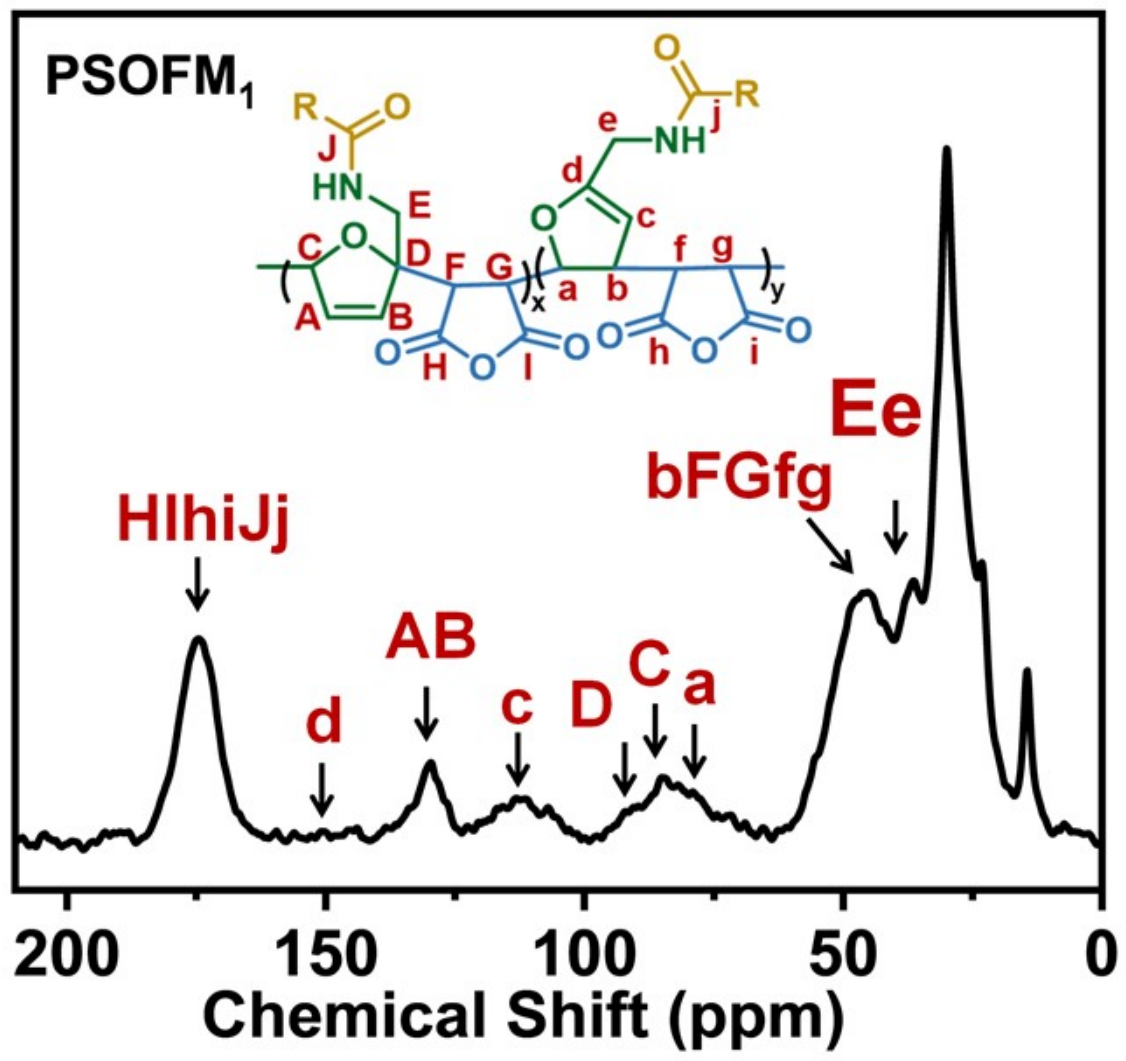

Figure S7. Solid-state ^{13}C NMR spectrum of $PSOFM_1$.

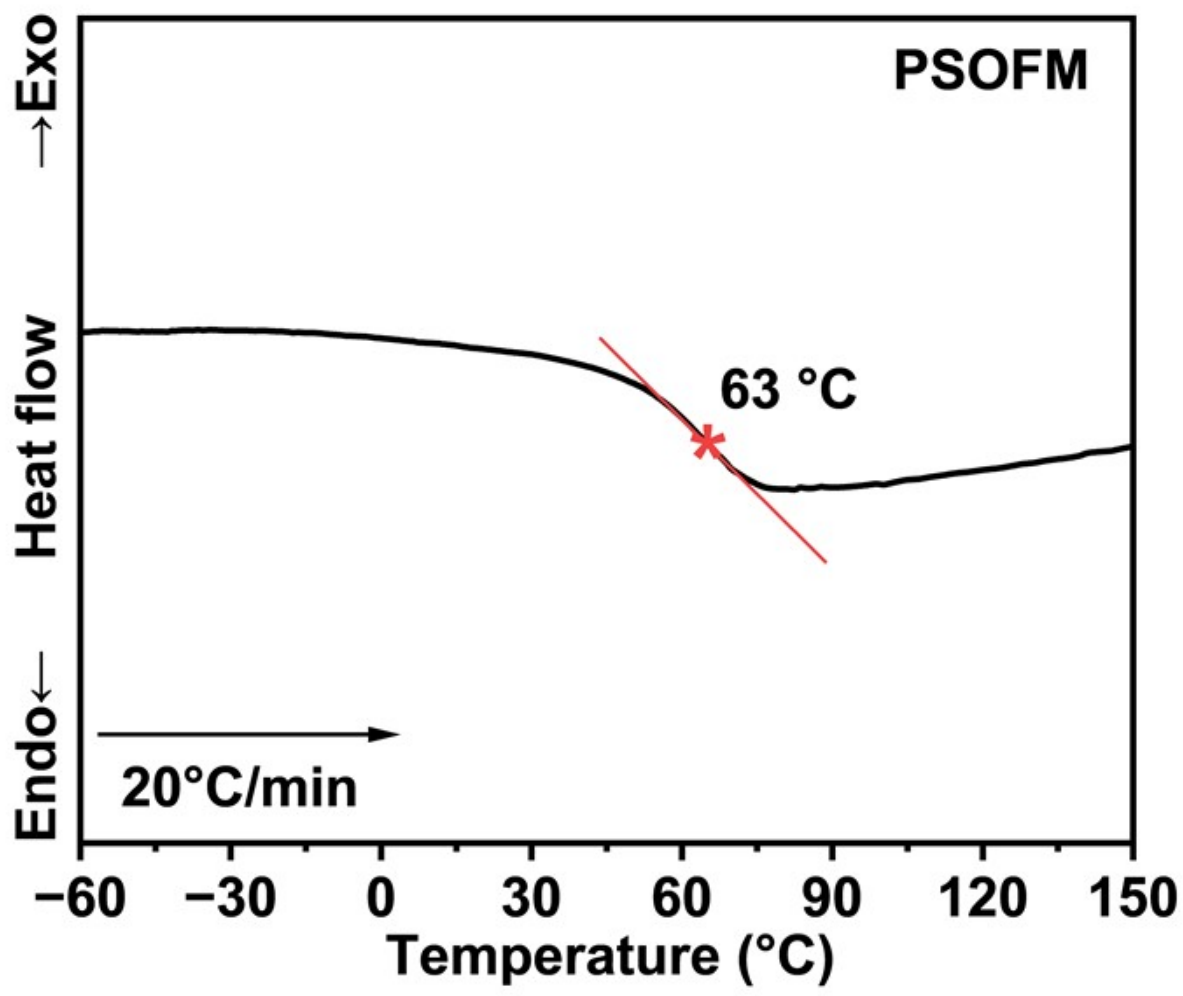

Figure S8. DSC curve of $PSOFM_1$.

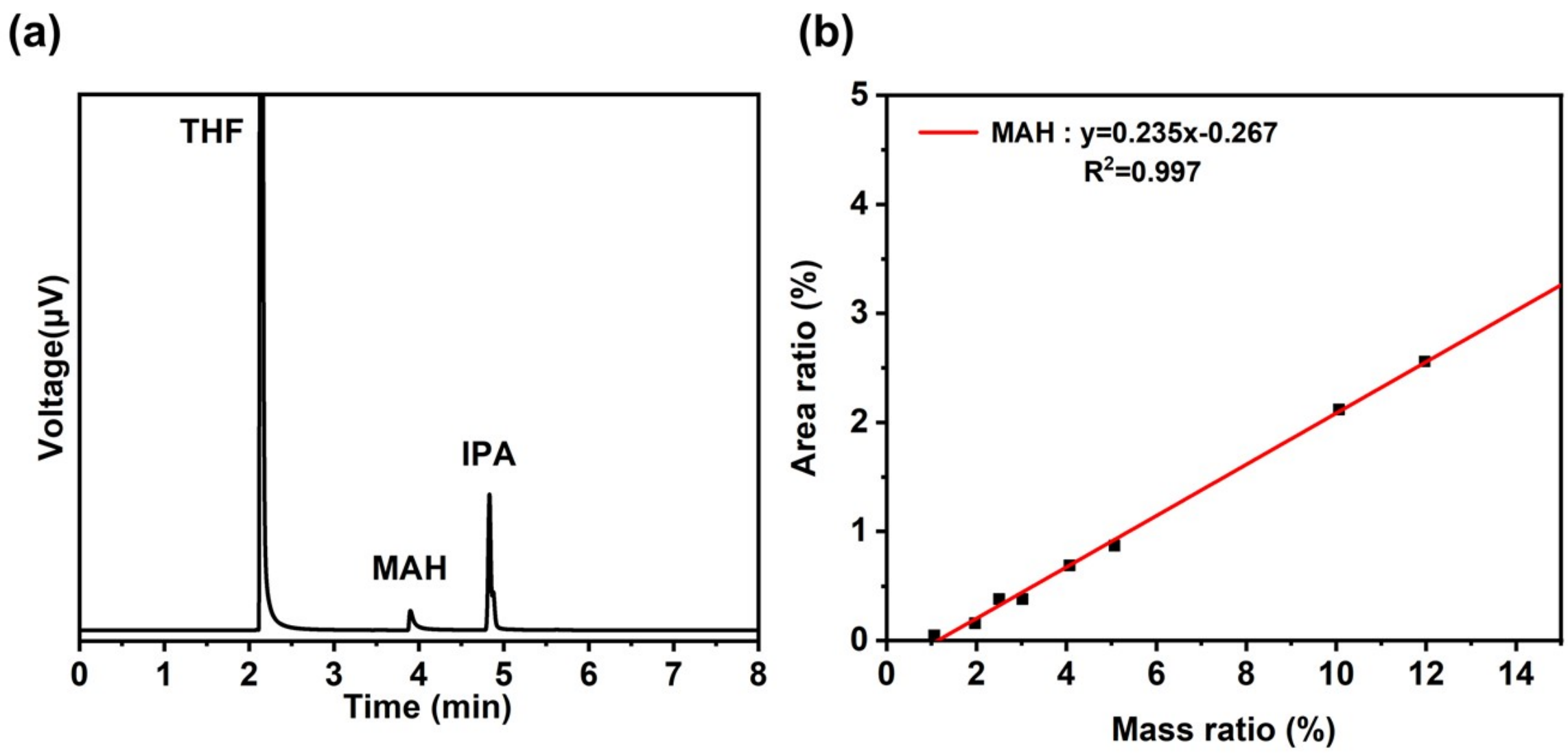

Figure S9. (a) GC chromatogram with MAH; (b) standard curve for MAH.

Analytical method: The content of MAH was determined using an internal standard method, with IPA serving as both the solvent and the internal standard. The GC temperature program was set as follows: the injector and detector temperatures were set at 250°C and 260°C, respectively, and the column temperature was set at 50°C. The column temperature was held at 50°C for 1 min, then raised to 110°C at a rate of 8℃/min and held for an additional 1 min. Under this program, both MAH and the internal standard exhibited well-defined chromatographic peaks and were easily distinguished. MAH eluted at approximately 3.9 min, while IPA eluted at around 4.8 min.

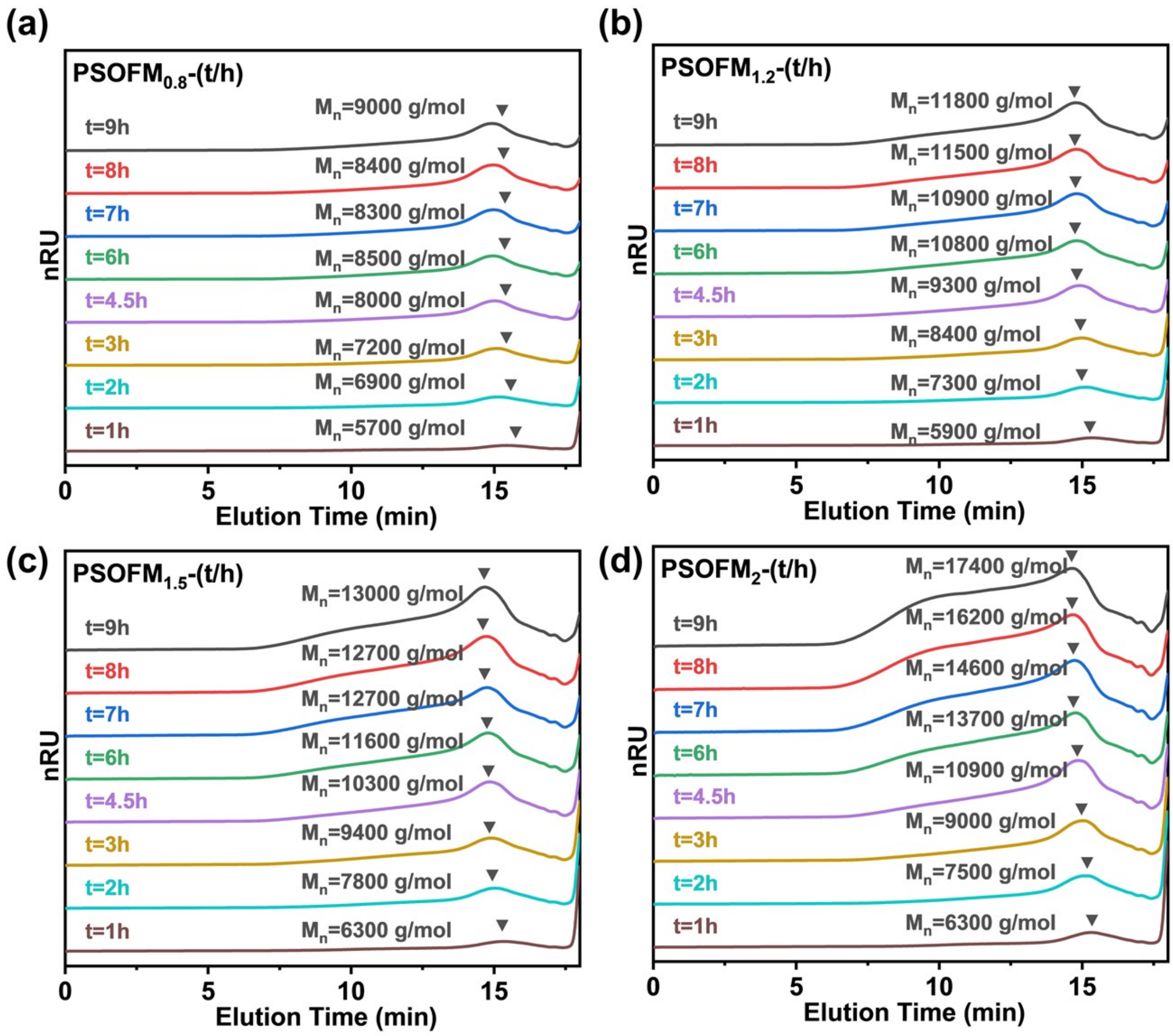

Figure S10. GPC curves of: (a) $PSOFM_{0.8}$; (b) $PSOFM_{1.2}$; (c) $PSOFM_{1.5}$; (d) $PSOFM_2$.

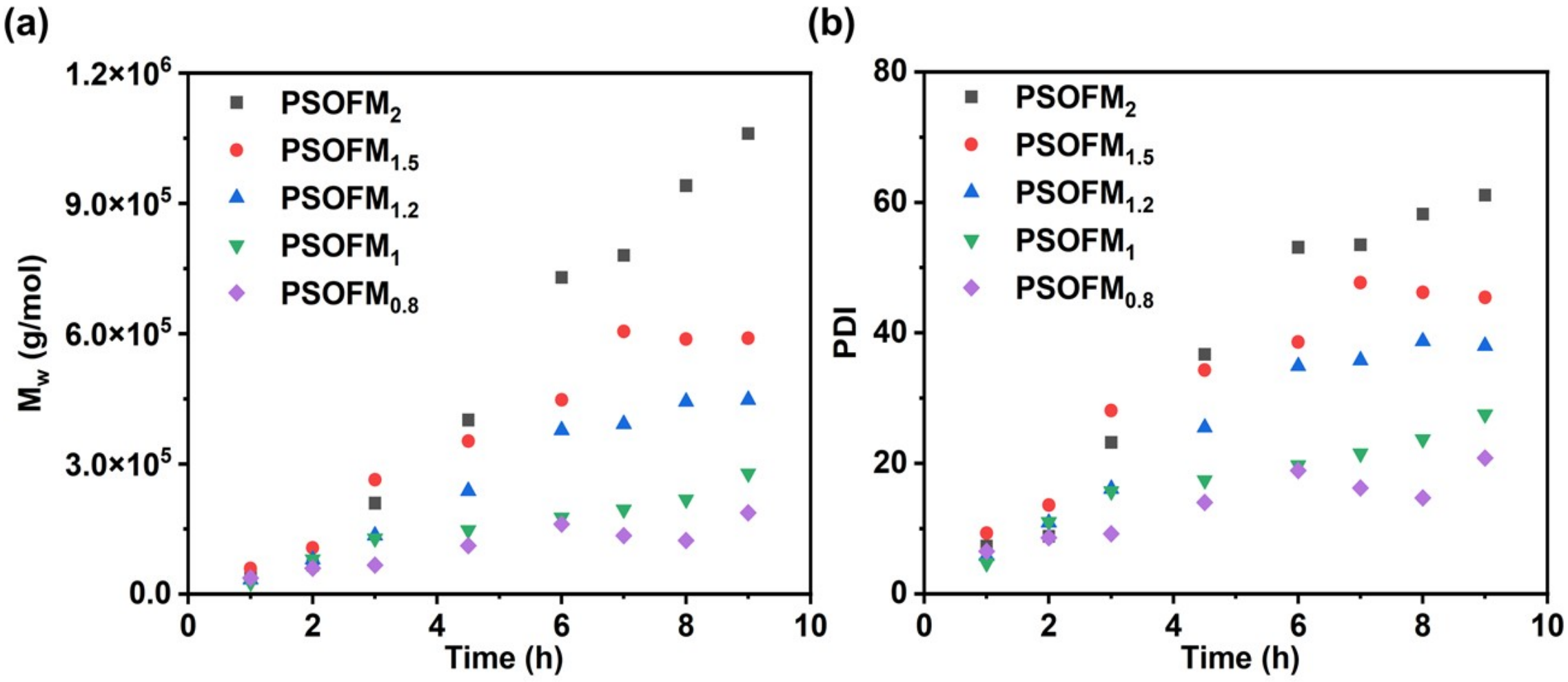

Figure S11. $PSOFM_a$: (a) M_w vs. time; and (b) PDI vs. time.

The samples in this work are designated as $PSOFM_a$ -variable. In this naming system, a = n(MAH)/n(SO-FA) (mol/mol); in the initiator type field, "A" denotes AIBN and "B" denotes BPO. Isoamyl acetate was used as the solvent in all experiments. The data of yield was obtained by calculating the precipitation using a mixed solvent of tetrahydrofuran/n-hexane (m/m) = 2/5.

Table S1. Copolymer composition of PSOFM at different monomer feed ratios.

Samples	Formula	elemental analysis		
		O (%)	N (%)	Content1(MAH) (wt%)
$PSOFM_2$	T = 75 °C; t=9h; C = 20%; AIBN-5%.	23.7%	2.6%	36%
$PSOFM_{1.5}$		25.0%	2.9%	37%
$PSOFM_{1.2}$		25.7%	2.9%	39%
$PSOFM_1$		25.1%	3.0%	38%
$PSOFM_{0.8}$		24.2%	2.9%	36%

Table S2. Influence of MAH/SO-FA monomer molar feed ratios

Samples	Formula	t/h	Yield	MAH conversion	M_n (g/mol)	M_w (g/mol)	PDI
$PSOFM_2$-(t/h)	T = 75 °C; C = 20%; AIBN-5%.	1	15%	22%	6300	45700	7.3
		2	25%	36%	7500	66000	8.8
		3	31%	50%	9000	209500	23.2
		4.5	52%	61%	10900	401000	36.7
		6	64%	66%	13700	729400	53.1
		7	72%	67%	14600	780300	53.5
		8	79%	70%	16200	940900	58.2
		9	79%	74%	17400	1060800	61.1
$PSOFM_{1.5}$-(t/h)	T = 75 °C; C = 20%; AIBN-5%.	1	——	22%	6300	58600	9.3
		2	——	51%	7800	106400	13.6
		3	17%	65%	9400	263500	28.1
		4.5	21%	66%	10300	352000	34.3
		6	36%	69%	11600	447200	38.6
		7	41%	73%	12700	604600	47.7
		8	60%	73%	12700	587500	46.2
		9	62%	77%	13000	589400	45.4
$PSOFM_{1.2}$-(t/h)	T = 75 °C;	1	——	28%	5900	33700	5.8

Samples	Formula	t/h	Yield	MAH conversion	M_n (g/mol)	M_w (g/mol)	PDI
	C = 20%;	2	——	60%	7300	79700	10.9
	AIBN-5%.	3	18%	67%	8400	135000	16.1
		4.5	20%	67%	9300	237600	25.5
		6	24%	75%	10800	377500	34.9
		7	28%	75%	10900	391900	35.8
		8	32%	76%	11500	443500	38.7
		9	41%	77%	11800	447700	38.0
$PSOFM_1$-(t/h)	T = 75 °C; C = 20%; AIBN-5%.	1	8%	45%	5700	26600	4.7
		2	9%	61%	7300	80000	11.0
		3	13%	70%	6200	128400	15.7
		4.5	20%	70%	6500	147400	17.4
		6	32%	73%	9000	176500	19.7
		7	40%	73%	9100	194800	21.5
		8	56%	75%	9200	217900	23.7
		9	57%	77%	10100	277900	27.5
$PSOFM_{0.8}$-(t/h)	T = 75 °C; C = 20%; AIBN-5%.	1	9%	54%	5700	37100	6.5
		2	11%	60%	6900	59400	8.6
		3	11%	66%	7200	66800	9.2
		4.5	19%	69%	8000	111600	14.0
		6	33%	72%	8500	161300	18.9
		7	32%	~100%	8300	134600	16.2
		8	37%	~100%	8400	123700	14.7
		9	36%	~100%	9000	187400	20.8

Note: The conversion is denoted by "—" because, in a mixed solvent of THF/n-hexane (2/5 by mass), no precipitation occurs, and the product remains fully dissolved in the solvent system.

Table S3. Influence of various reaction conditions.

(a) The amount of initiator was used as the variable.

Samples	Formula	Yield	M_n (g/mol)	M_w (g/mol)	PDI
$PSOFM_1$-A1	T = 75 °C;	23%	9600	172200	18.0
$PSOFM_1$-A3	C = 20%;	28%	9400	177100	18.8
$PSOFM_1$-A5	t = 8h.	56%	9200	217900	23.7
$PSOFM_1$-A5-2.5h	T = 80 °C;	17%	7600	87900	11.5
$PSOFM_1$-B5-7h	C = 20%.	19%	3100	4100	1.3

(b) The monomer concentration was used as the variable.

Samples	Formula	Yield	M_n (g/mol)	M_w (g/mol)	PDI
$PSOFM_1$-C20	T = 75 °C;	56%	9200	217900	23.7
$PSOFM_1$-C15	AIBN-5%;	30%	8200	141000	17.2
$PSOFM_1$-C10	t = 8h.	20%	6500	57400	8.8

(c) The temperature was used as the variable.

Samples	Formula	Yield	M_n (g/mol)	M_w (g/mol)	PDI

**$PSOFM_1$-T70(9h)**		**29%**	**10000**	**247200**	**24.8**
**$PSOFM_1$-T75(4.5h)**		**20%**	**6500**	**147400**	**17.4**
**$PSOFM_1$-T80(2.5h)**		**17%**	**7600**	**87900**	**11.5**
**$PSOFM_{1.2}$-T70(9h)**		**43%**	**10400**	**285400**	**27.4**
**$PSOFM_{1.2}$-T75(4.5h)**	**C = 20%; AIBN-5%.**	**20%**	**9300**	**237600**	**25.5**
**$PSOFM_{1.2}$-T80(2.5h)**		**18%**	**8100**	**130400**	**16.0**
**$PSOFM_2$-T70(9h)**		**62%**	**12500**	**497800**	**39.8**
**$PSOFM_2$-T75(4.5h)**		**52%**	**10900**	**401000**	**36.7**
**$PSOFM_2$-T80(2.5h)**		**35%**	**9000**	**204100**	**22.7**

In this part, MAH and different amides were dissolved in IPA at an equimolar ratio, with a monomer concentration of 20% and an initiator dosage of 5 wt% based on monomer weight. The copolymerization was conducted at 75 °C for 8 h, synthesizing the corresponding copolymers, which are designated as PSFM, POFM and PLFM, respectively.

Table S4. Influence of fatty chains.

Samples	Mn	Mw	PDI
**PSFM**	**2600**	**3100**	**1.23**
**POFM**	**3200**	**4200**	**1.32**
**PLFM**	**3600**	**4800**	**1.36**

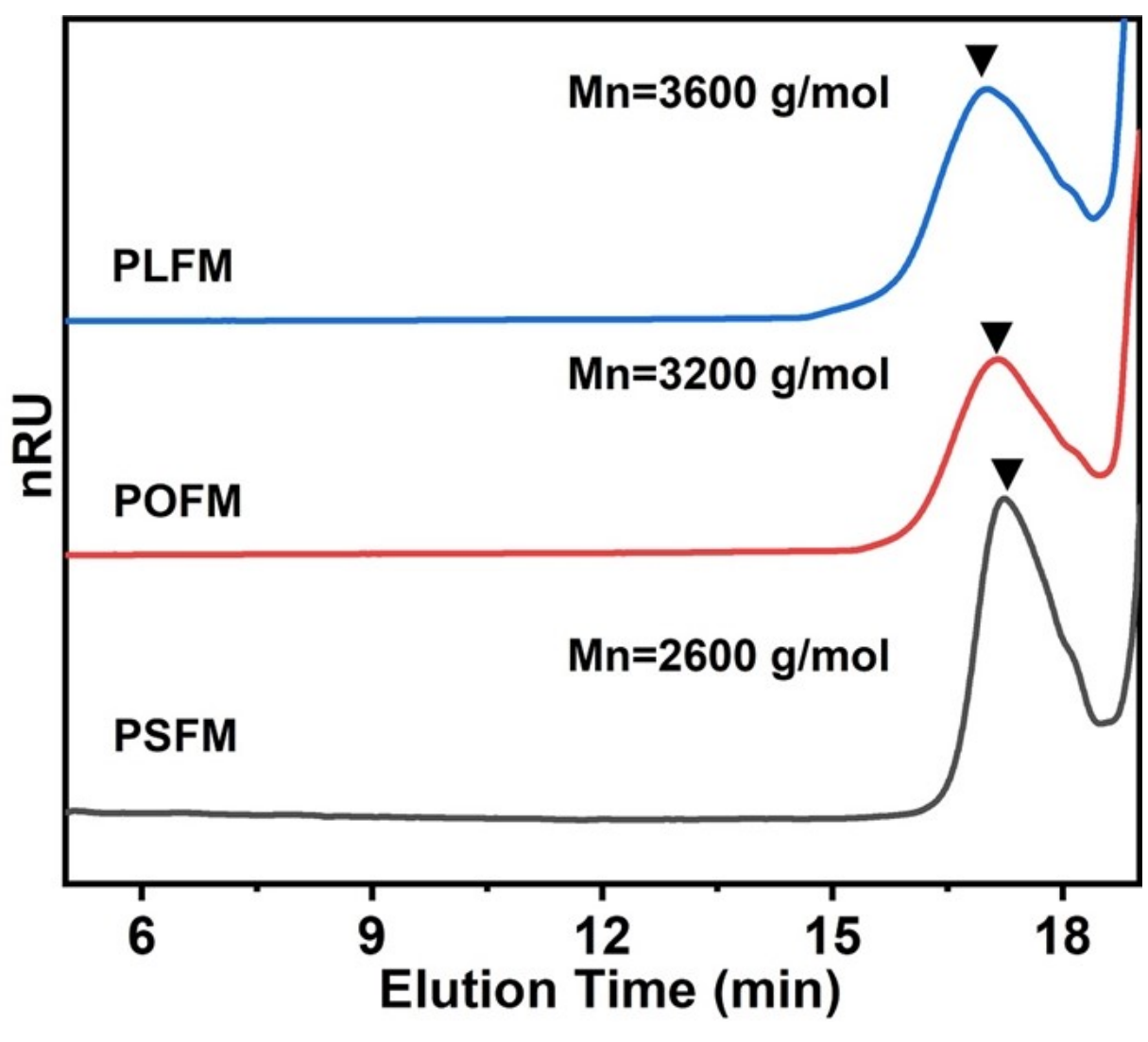

Figure S12. GPC curves of PSFM, POFM and PLFM.
